# New Insights into Molecular Organization of Human Neuraminidase-1: Transmembrane Topology and Dimerization Ability

**DOI:** 10.1038/srep38363

**Published:** 2016-12-05

**Authors:** Pascal Maurice, Stéphanie Baud, Olga V. Bocharova, Eduard V. Bocharov, Andrey S. Kuznetsov, Charlotte Kawecki, Olivier Bocquet, Beatrice Romier, Laetitia Gorisse, Maxime Ghirardi, Laurent Duca, Sébastien Blaise, Laurent Martiny, Manuel Dauchez, Roman G. Efremov, Laurent Debelle

**Affiliations:** 1 Laboratoire Signalisation et Récepteurs Matriciels (SiRMa), UMR CNRS 7369 Matrice Extracellulaire et Dynamique Cellulaire (MEDyC), Université de Reims Champagne Ardenne (URCA), UFR Sciences Exactes et Naturelles, Reims, France; 2 Plateau de Modélisation Moléculaire Multi-échelle, UFR Sciences Exactes et Naturelles, URCA, Reims, France; 3 M. M. Shemyakin and Yu. A. Ovchinnikov Institute of Bioorganic Chemistry, Russian Academy of Sciences, Moscow, Russia; 4 Higher School of Economics, Myasnitskaya ul. 20, 101000 Moscow, Russia

## Abstract

Neuraminidase 1 (NEU1) is a lysosomal sialidase catalyzing the removal of terminal sialic acids from sialyloconjugates. A plasma membrane-bound NEU1 modulating a plethora of receptors by desialylation, has been consistently documented from the last ten years. Despite a growing interest of the scientific community to NEU1, its membrane organization is not understood and current structural and biochemical data cannot account for such membrane localization. By combining molecular biology and biochemical analyses with structural biophysics and computational approaches, we identified here two regions in human NEU1 - segments 139–159 (TM1) and 316–333 (TM2) - as potential transmembrane (TM) domains. In membrane mimicking environments, the corresponding peptides form stable α-helices and TM2 is suited for self-association. This was confirmed with full-size NEU1 by co-immunoprecipitations from membrane preparations and split-ubiquitin yeast two hybrids. The TM2 region was shown to be critical for dimerization since introduction of point mutations within TM2 leads to disruption of NEU1 dimerization and decrease of sialidase activity in membrane. In conclusion, these results bring new insights in the molecular organization of membrane-bound NEU1 and demonstrate, for the first time, the presence of two potential TM domains that may anchor NEU1 in the membrane, control its dimerization and sialidase activity.

Sialidases, or neuraminidases, represent a family of exoglycosidases that remove terminal sialic acid residues from glycoproteins, glycolipids and oligosaccharides^1^. These enzymes are widely distributed and found in viruses, protozoa, bacteria, fungi, and vertebrates^2^. The human sialidase family contains four members: the lysosomal NEU1 (Swiss-Prot:Q99519), cytosolic NEU2, and membrane-bound NEU3 and NEU4^1^,^3^. Each sialidase presents distinct substrate specificity and subcellular localization^4^. All of them are assumed to share the typical β-propeller structure organized in six blades, each composed of four antiparallel β-sheets, and the highly conserved motifs for their catalytic activity: the three Arg residues involved in binding of sialic acids, a Tyr/Glu nucleophile motif and an Asp residue serving as proton acceptor during the catalysis^5^. To date, NEU1 has been the most studied sialidase, notably because NEU1 deficiency is linked to genetic diseases, sialidosis and galactosialidosis^6^. Sialidosis, caused by a NEU1 deficiency, is a fulminant disease that develops before birth; the patients are stillborn or die soon after birth^7^. In contrast, galactosialidosis is caused by protective protein/cathepsin A (PPCA) deficiency with a combined secondary near-complete deficiency of NEU1. The biochemical hallmark of both lysosomal storage disorders is the progressive accumulation of sialylated glycoproteins, glycopeptides and oligosaccharides in lysosomes of many cell types, as well as excretion of sialyloligosaccharides in body fluids. The involvement of NEU1 in lysosomal diseases clearly demonstrates that NEU1 functions as a lysosomal enzyme and is supported by its optimal acidic pH for catalysis^4^.

Nevertheless, a plasma membrane-bound NEU1, controlled by the phosphorylation of its C-terminus, has been also reported^8^. At the plasma membrane, NEU1 has been shown to be required for signal transduction and elastogenesis through the elastin receptor complex^9^,^10^,^11^ and to be involved in the modulation of insulin receptor signaling^12^,^13^, regulation of integrin beta 4^14^, TLR4^15^, Trk A^16^, PDGF-BB and IGF receptors^17^, EGF and MUC1 receptors^18^ and more recently CD31^19^. Consequently, NEU1 now emerges not only as a catabolic enzyme but also as a key actor involved in cell signaling regulation^20^. Despite a growing interest of the scientific community to NEU1, its organization and dynamics in membrane are not understood. It is established that NEU1 is routed to the lysosomal compartment where it forms a multi-enzyme complex with PPCA and β-galactosidase^7^. PPCA acts as a chaperone for NEU1 preventing its premature oligomerization^21^ and PPCA/NEU1 association is required for proper addressing to the lysosome and for its sialidase activity^22^. How NEU1 translocates to the plasma membrane in association with PPCA, or not, is not known. In addition, its membrane topology is still puzzling. Indeed, NEU1 sialidase activity can be measured at the cell surface indicating that its active site would be extracellular. Besides, the phosphorylation of its C-terminus indicates that NEU1 contains an intracellular domain. Nevertheless, no current sialidase molecular model can account for these two features. Given the critical roles played by membrane-bound NEU1, the present study was designed to characterize the organization and assembly of human NEU1 in membrane using a combination of biology-based, biophysical and computational approaches.

## Results

### Analysis of amino acid sequence of human NEU1 reveals putative TM regions

**Figure 1:**
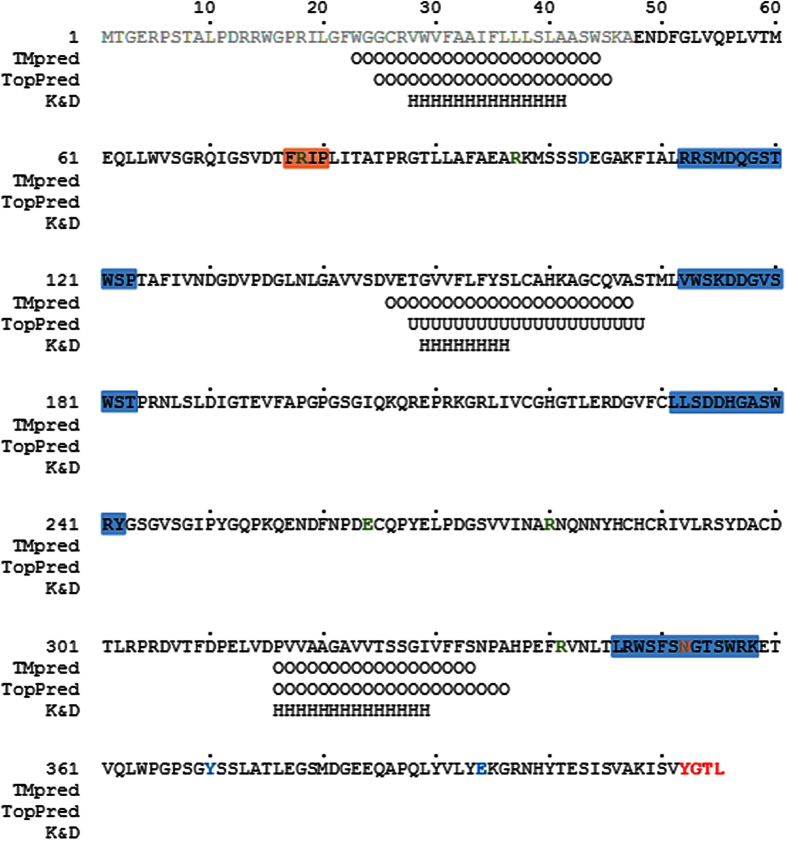
Sequence analysis of human NEU1. Light gray, putative signal peptide. Bold red, internalization signal. Blue boxes, BNR repeats. Red box, FRIP motif. Orange bold, confirmed glycosylation site. Bold blue, active site residues. Bold green, residues interacting with the substrate. TMpred, prediction of transmembrane domain by the TMpred tool. TopPred, topology prediction of membrane protein by the TopPred tool. Positions predicted in a TM domain with a high level of confidence are marked ‘O’, those with a lower level are maked ‘U’. K&D, hydrophobic sequence (score > 1.5) predicted using the Kyte and Doolittle ProtScale tool. Positions predicted as hydrophobic are labeled ‘H’.

The human NEU1 sequence (Q99519) was subjected to bioinformatic analysis in order to delineate potential transmembrane (TM) domains. Three prediction algorithms (TMpred, TopPred, ProtScale) were used and underlined that the protein sequence contains hydrophobic stretches that could possibly cross the plasma membrane (Fig. 1). All the algorithms evidenced three possible domains: one in the putative signal peptide sequence^23^, one between bacterial neuraminidase repeats (BNR) 1 and 2 and another one in the C-terminal part of NEU1. Analysis of sequence hydrophobicity identified these three regions as rather hydrophobic, although according to TopPred algorithm, the second TM fragment (residues 148–168, NEU1/TM1) is less probable than the other two (25–45, NEU1/TM0 and 316–336, NEU1/TM2).

### A pool of NEU1 is present at the plasma membrane of COS-7 cells in the absence of PPCA

**Figure 2:**
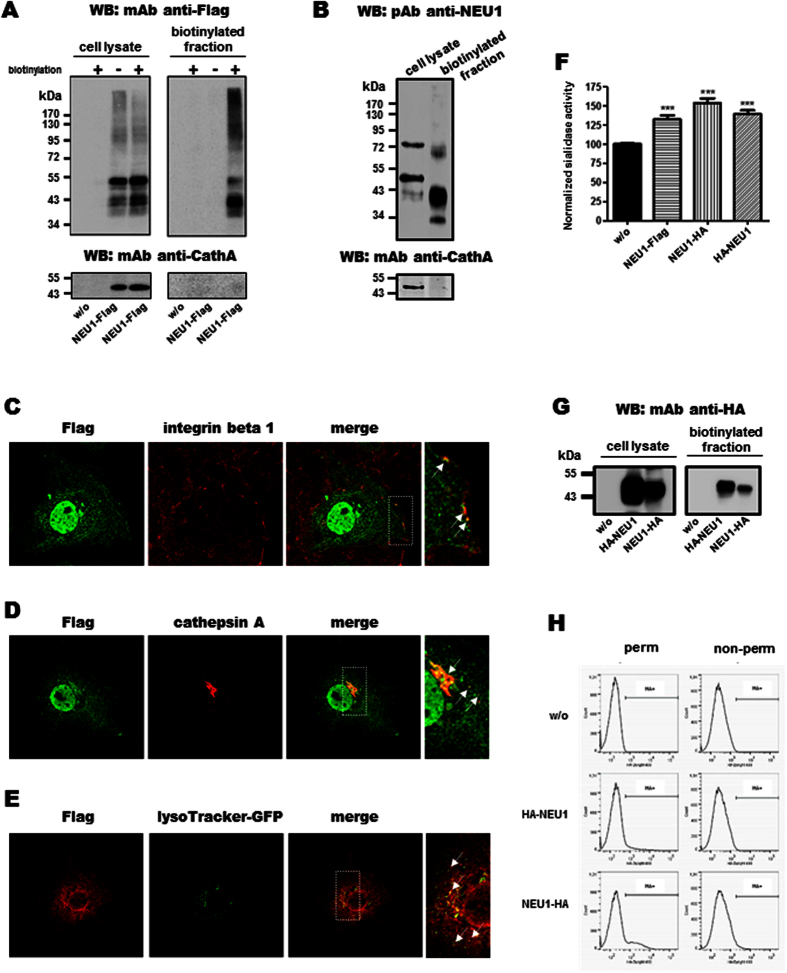
NEU1 is present at the plasma membrane in the absence of PPCA. (**A**,**B**) Western blot on the plasma membrane proteins (biotinylated fraction) of (**A**) COS-7 cells co-transfected with NEU1-Flag and PPCA (1:2) and probed with a mouse monoclonal anti-Flag or -cathepsin A antibody, or (**B**) human macrophages, endogenously expressing both NEU1 and PPCA, and probed with a rabbit polyclonal anti-NEU1 and mouse monoclonal anti-cathepsin A antibody. A representative pattern of (**A**) three and (**B**) two experiments is shown. (**C**–**E**) Confocal images of (**C**) NEU1-Flag and integrin beta 1, or (**D**) NEU1-Flag and cathepsin A, or (**E**) NEU1-Flag and lysosome distribution in permeabilized COS-7 cells co-transfected with NEU1-Flag and PPCA (1:2). (**F**) Sialidase activity measured from adherent COS-7 cells co-transfected with NEU1-Flag, NEU1-HA, or HA-NEU1 and PPCA (1:2). Plates were incubated with 200 μM 2-O-(p-nitrophenyl)-α-d-N-acetylneuraminic acid substrate in MES 20 mM, pH 4.5. Sialidase activity was measured at 405 nm and results expressed as mean ± SEM of 3 independent experiments, each run in duplicate and normalized to the control (w/o); e.g. cells transfected with PPCA and empty vector. ***p < 0.001. (**G**) Western blot on the biotinylated fraction of COS-7 cells co-transfected with HA-NEU1 or NEU1-HA and PPCA (1:2), and probed with a rabbit monoclonal anti-HA antibody. A representative pattern of three experiments is shown. (**H**) Detection by flow cytometry of HA-NEU1 or NEU1-HA co-expressed in COS-7 cells with PPCA (1:2). Cells were permeabilized, or not, by 0.1% saponin and both constructs detected using a Dylight 488-conjugated mouse monoclonal anti-HA antibody. A representative pattern of three experiments is shown.

Wild-type human NEU1-Flag was co-expressed in COS-7 cells together with human PPCA, which has been shown to be required for correct folding, compartmentalization and catalytic activation of NEU1 in lysosomes^6^,^23^. Transient expression of NEU1-Flag gives rise to multiple protein species of molecular weight between 40 and 55 kDa due to differential glycosylation of NEU1^24^ (Fig. 2A). Cell surface protein labelling with the non-permeable reagent EZ-Link^®^ sulfo-NHS-LC-biotin allowed the recovery of NEU1-Flag in the biotinylated plasma membrane fraction but not of PPCA (Fig. 2A). Similar results were obtained in human macrophages that endogenously express both NEU1 and PPCA (Fig. 2B). In addition, monomers and presumably dimers of NEU1 were recovered (Fig. 2A,B). Immunofluorescence experiments performed on permeabilized COS-7 cells showed intracellular staining of NEU1-Flag and staining at the cell membrane as attested by some co-localization areas with integrin beta 1 (Fig. 2C). When looking for PPCA, co-localization areas with NEU1-Flag were observed inside the cells but not at the plasma membrane (Fig. 2D). Finally, some co-localization areas were also observed with lysosomes (Fig. 2E).

Strikingly, detection of NEU1-Flag by anti-Flag antibodies could only be achieved in permeabilized cells (Fig. 2C–E) and similar results were obtained by flow cytometry with two other constructs, HA-NEU1 and NEU1-HA, in which the HA tag was located at the N- or C-terminus of NEU1, respectively. Although these two functionally active constructs (Fig. 2F) were recovered in the plasma membrane fraction (Fig. 2G), no labelling could be observed in non-permeabilized cells (Fig. 2H). In contrast, prior permeabilization of COS-7 cells increased the percentage of HA-positive cells (Fig. 2H).

Taken together, these results demonstrate that monomers and presumably dimers of NEU1 are present at the membrane, in the absence of any detectable association with PPCA, and suggest that both extremities of NEU1 are oriented towards the cytosol.

In order to shed light on the molecular structural details of NEU1 interaction within the membrane and its ability to dimerize in the membrane-bound state, we focused on the most probable TM region (TM2) ^316^PVVAAGAVVTSSGIVFFS^333^ (NEU1/TM2) delineated based on the sequence analysis (Fig. 1). The second putative TM region ^148^TGVVFLFYSLCAHKAGCQVAS^168^ (NEU1/TM1) was also studied and the third one (NEU1/TM0) omitted because of its localization within the predicted signal peptide sequence. We explored their conformational preferences and potency to self-association in membrane-mimicking environment using a combination of biophysical and simulation methods. First, direct experimental structural data were obtained in membrane mimicking media using NMR spectroscopy and circular dichroism (CD). This was done for the produced ^15^N-labeled recombinant fragments R^305^DVTFDPELVDPVVAAGAVVTSSGIVFFSNPAHPEFR^341^ (named as hNEU1/TMS2) and D^130^GDVPDGLNLGAVVSDVETGVVFLFYSLCAHKAG*S*QVASTMLV WSKDDGVS^180^ (named as hNEU1/TMS1) encompassing the predicted helical TM segments (underlined residues with adjacent regions being potentially also transmembrane). The results helped in refinement of the boundaries of TM segments – for this purpose the peptide sequences were taken rather longer than it is required to span the membrane. Then, the peptides were subjected to molecular dynamics (MD) simulations in model membranes - explicit dipalmitoylphosphatidylcholine (DPPC) and palmitoyloleoylphosphatidylcholine (POPC) bilayers in order to probe structural stability of the proposed α-helical conformation in heterogeneous lipid-water milieu. Finally, because the ability of Neu-1/TMS2 to associate in membrane was shown by NMR analysis, we explored spatial structures of putative dimers formed by NEU1/TM2. This was done using two independent approaches: (i) MD simulations in DPPC bilayer; (ii) PREDDIMER calculations followed by MD relaxation and free energy calculations in POPC (see Methods).

### The regions 139–159 and 316–333 of NEU1 form stable helical structures in DPC environment and homodimerize

**Figure 3:**
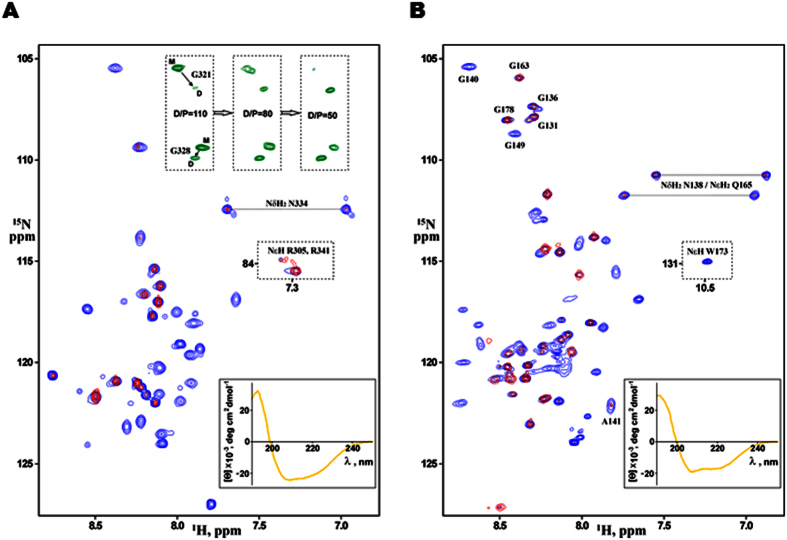
NMR and CD spectra of hNEU1/TMS2 and hNEU1/TMS1 fragments in membrane-mimicking environment. (**A**,**B**) Overlaid NMR spectra in blue and red (the ^1^H/^15^N-HSQC and CLEANEX NMR spectra, respectively) of (**A**) NEU1/TMS2 and (**B**) NEU1/TMS1 fragments embedded into DPC micelles at the detergent/protein molar ratio (D/P) of 200. Amide cross-peak dispersion in the ^1^H/^15^N-HSQC spectra is typical for membrane helical proteins^62^. Amount of water-exposed amide groups monitored by the CLEANEX spectra suggests that about 20 residues of each fragment are located within the micelle. According to NOE connectivities, four transmembrane glycine residues G^321^/G^328^ and G^140^/G^149^ were identified in NEU1/TMS2 and NEU1/TMS1, respectively. In the top of panel **A**, sequential NMR spectra in *green* within three boxes present the glycine regions of the ^1^H/^15^N-TROSY NMR spectra acquired for NEU1/TMS2 embedded into micelles at D/P varied from 110 to 50. The doubling of the amide group cross-peaks of the transmembrane G^321^ and G^328^ residues indicates a slow monomer-dimer (or oligomer) transition of NEU1/TMS2 in the membrane-mimicking environment. At D/P = 200 some amide cross peaks of both fragments have a minor component presumably due to slow *cis/trans* transitions of V^133^-P^134^, D^310^-P^311^ and H^337^-P^338^ peptide bonds in the flexible solvent-exposed N- and C-termini. In the case of NEU1/TMS1, a signal broadening is observed due to intermediate conformational exchange processes or/and some oligomerization. In the bottom of panels A and B, the CD spectra, shown in *yellow*, reveal the 50% and 46% of the α-helical structure of NEU1/TMS2 and NEU1/TMS1, respectively.

**Figure 4:**
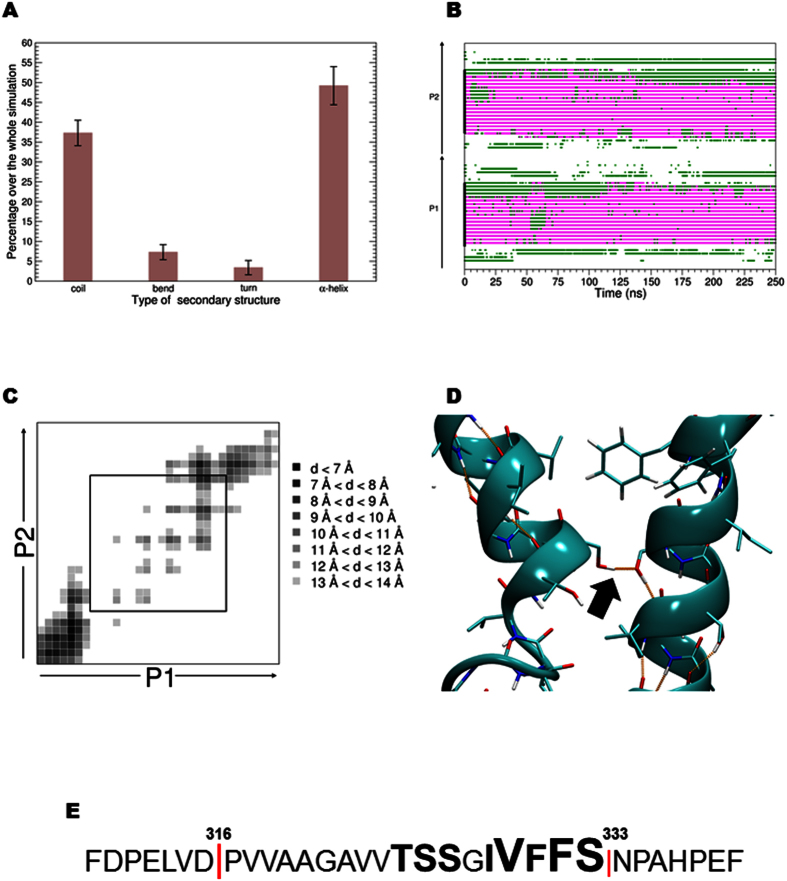
Monitoring of local secondary structure and contacts between NEU1/TM2 peptides. (**A**) Average secondary structure and standard deviations were computed for the twenty-four independent simulations performed in DPPC. (**B**) Time evolution of the secondary structure corresponding to the simulation with a starting conformation angle equal to 30 degrees. Green indicates residues in a turn structure, pink in an α-helix structure and white in a coil structure. The nature of the peptides is indicated on the y-axis (P1 or P2). (**C**) Contact map obtained over the last 10 ns of the trajectory corresponding to a starting conformation angle of 30 degrees. For each pair of residues, the average smallest atomic distance was computed over the last 1000 snapshots of the trajectory. The contacts displayed in the central square correspond to contacts within the membrane. (**D**) Snapshot extracted from a simulation highlighting the presence of a H-bond between two serine residues (S326-S327). (**E**) Key-residues (bold letters) involved in contacts between the two helices according to the analysis of the twenty-four contact maps.

The ^1^H/^15^N-heteronuclear NMR spectra clearly demonstrated that, being embedded into membrane-mimicking n-dodecylphosphocholine (DPC) micelles, both hNEU1/TMS2 and hNEU1/TMS1 fragments were mostly folded into a helical conformation having the characteristic dispersion from 7.5 ppm to 9.5 ppm for the ^1^H-chemical shifts of the amide groups (Fig. 3). In both cases, CLEANEX experiment revealed that about 20 amide protons are less water-accessible than the adjacent residues, probably due to effective shielding from solvent caused by hydrogen-bonding within the TM helix and/or by insertion of this region into the DPC micelle. This observation was in a reasonable agreement with the CD data and MD simulations (see below) indicating about 45–50% of the α-helical structure (Figs 3 and 4A). Increasing the detergent hydrocarbon tails from 12 up to 16 carbons (by using Fos-Choline-16 instead of DPC) does not essentially change the pattern of solubilization in the membrane-mimicking environment for both fragments although some local changes are however observed in both TM helix structures (Supplementary Fig. S1). According to sequential NOE connectivities observed in the ^1^H/^15^N-NOESY-HSQC spectra, four transmembrane glycine residues G^321^/G^328^ and G^140^/G^149^ were unambiguously identified in NEU1/TMS2 and NEU1/TMS1, respectively. Nevertheless, a weak cross-peak was observed in the CLEANEX spectrum for G^328^ amide group, indicating facilitated exchanges with water protons that could be related either to water penetration into the ^325^TSSG^328^ polar patch on the surface of the proposed TM helix that can be inherent to serine-rich TM motifs^25^ and/or to local helix bending resulting in exposure of this region from micelle to bulk water. In addition, the preliminary analysis of the NOE connectivities revealed tentative regions of the helical NEU1 TM segments as L^313^-S^333^ and L^139^-A^159^.

The ^1^H/^15^N-TROSY NMR spectrum of hNEU1/TMS2 embedded in DPC micelles at D/P = 110 displayed an additional set of cross-peaks as compared with the signals expected based solely on the amino acid sequence (three boxes in the top right corner of Fig. 3A). These additional cross-peaks were not detected in the spectrum at D/P = 200. According to biotinylation results, the new set of cross-peaks could result from self-association (presumably dimerization) of NEU1/TMS2 in the micelles saturated by the peptide. As the minimal distinguishable chemical shift difference between signals of two states in the ^1^H/^15^N-TROSY spectrum is about 20 Hz, the monomer-dimer transition is supposed to be a slow process (on the millisecond timescale). The dimer (or oligomer) population was increased rapidly with decrease of D/P values as typical for weak dimerized helical TM domains of different proteins^26^,^27^. Unfortunately, at D/P < 100, the NEU1/TMS1 sample was unstable and precipitated perhaps due to oligomerization. D/P-dependent occupancy of the states also implies that the fragments are associated with the micelle. Thus, the acquired NMR and CD data proved that both NEU1/TMS2 and NEU1/TMS1 fragments penetrate into the hydrophobic core of the membrane-mimicking DPC micelle and fold into a helical conformation.

These direct experimental structural data provide a solid ground for molecular modeling of the both TM segments of NEU1. Thus, it became possible to precise their boundaries. While the peptide NEU1/TM2 remains the same as predicted based on the sequence analysis (see above), serious corrections were made for NEU1/TM1 segment. According to NMR data, the most probable membrane-spanning part is the sequence 139–159, whereas the predictions prompted the segment 148–158. One favorable consequence of this change is exclusion of K161 from TM region. On the other hand, two rather polar residues – D145 and E147 – appear in the TM domain. Nevertheless, the aspartate and glutamate residues are occasionally found in TM segments of different proteins, e.g. as gain-of-function mutations, which probably enhance the membrane receptor dimerization^28^,^29^. Stability and membrane insertion of the NEU1/TM1 helix would depend on the protonation state of the side chain carboxyl. Since D145 and E147 are located quite in the central TM part of NEU/TM1, which is buried from the bulk water, their pKa values should increase, resulting in protonation of the side chain carboxyls even under physiological conditions (at pH ~7)^30^. Indeed, our NMR data revealed that the identified NEU1/TMS1 helix penetrates into the hydrophobic part of the micelle, suggesting protonation of D145 and E147 at pH 6.6. In the organism, the membrane surface potential, lipid composition of the cell membrane, ion concentration, and, possibly, extracellular domain conformation affect the pKa values of D145 and E147 and, thus, the stability and membrane insertion of the NEU1/TM1. Noteworthy, the inside of a lysosome where NEU1 is usually functioning is rather acidic (pH ~ 5)^4^. Hence, ionization state of these residues can represent a regulation level of the NEU1 activity.

The aforementioned results clearly demonstrate advantages of the present combined – experimental and theoretical – approach, especially in identification of relatively undistinguishable TM domains (like TM1) in proteins. Therefore, subsequent simulations were carried out for the peptides having TM cores 139–159 and 316–333.

### MD simulations predict that NEU1/TM2 preserves stable helical conformation in DPPC and POPC membranes and dimerizes

The use of MD simulations throughout this study had four aims: (i) check if TM helices were stable in a membrane-like environment; (ii) explore the ability of two individual peptides to self-associate in membrane; (iii) characterize TM dimers with respect to TM monomers in terms of helix-helix packing, free energy of dimerization and dimer stability; and (iv) correlate the predicted models for dimerization with MD data analysis. For these issues, various dimeric conformations were explored starting from different mutual dispositions of non-interacting NEU1/TM2 helices in POPC and DPPC membranes in coarse-grained (CG) and full-atomic representations, respectively. In the first case, massive rapid screening of the peptides’ configurational space was done. The objective here was to check whether the monomers stay isolated or associate. The results obtained in the course of 144 independent CG MD 1-μs trajectories clearly demonstrated efficient spontaneous dimerization of TM2 helices in lipid bilayer. Thus, in the vast majority of cases, stable dimers were formed already within first microseconds of CG MD (Supplementary Fig. S2A). Interestingly, after certain times, the dimers do not dissociate although at the adaptation stage both monomers and dimers were observed. Furthermore, most of the dimers were clustered in only four groups of structure with almost parallel packing of the helices but varying dimerization interfaces. As shown in Supplementary Fig. S2B, the most populated state revealed the helix packing similar to the lowest energy one obtained using independent all-atom simulations (the so-called model 2, see below). The accumulated rather large overall MD statistics (144 μs) strongly supports our hypothesis about dimerization of NEU1 TM2 segments. On the other hand, taking into account relatively short CG simulation times (1-μs trajectories), these results seem to be insufficient yet to elaborate precise 3D models of the dimers. This was further done using complementary all-atom techniques (see below). Nevertheless, the largest cluster of the dimers revealed an interface (residues S327, V330, F331) similar to that predicted *via* two independent all-atom MD techniques (see below).

In the first of them, twenty-four full-atomic MD runs in DPPC were performed for two TM2 helices with their main axes conserved parallel (Supplementary Fig. S3). The average content of α-helix was about 49% along the simulations (Fig. 4A). Analysis of time evolution of all the systems shows that the starting α-helical structure was very stable and did not break (Fig. 4B). Contact maps presenting the average smallest distance between residues were computed over the 250 nanoseconds of each simulation (Fig. 4C). These maps show that peptides get closer for particular angle values between the helices, namely 0°, 30°, 120°, and 330°. Moreover, the amino acids participating in contacts were mostly situated in the C-terminal region of the helix (Fig. 4C). Combining the results of the twenty-four simulations, the following amino acids were delineated as recurrent in the interaction interface between the two helices: T325, S326, S327, I329, V330, F331, F332, and S333 (Fig. 4E). Among these residues, S326 and S327 were evidenced as being able to form intermolecular hydrogen bonds (Fig. 4D).

### Sequence-based PREDDIMER calculations reveal two putative dimer structures for NEU1/TM2

**Figure 5:**
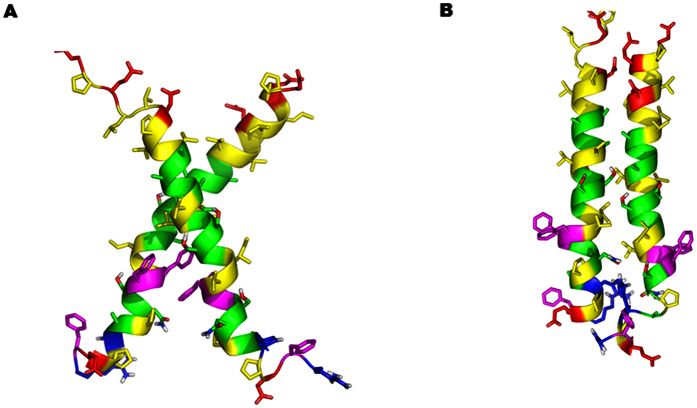
Side views of NEU1/TM2 dimer structures generated by the PREDDIMER software. NEU1/TM2 homodimer models 1 (top) and 2 (bottom). Average conformations obtained from the equilibrated part of MD trajectory are shown. The following scheme of residue coloring is employed: *green*, small and polar; *yellow*, aliphatic; *violet*, aromatic; *blue*, positively charged; *red*, negatively charged.

Sixteen possible conformations of a NEU1/TM2 (P316-S333) dimer were generated using three independent runs of PREDDIMER^31^ with varying TM sequence fragment length. Geometrical parameters like crossing angle and distance between the helix axes were inspected to determine different groups of conformers. As a result, nine groups were selected. Some of them included several structures from different starting geometries and others were represented by single structures. For further study, we took two structures representing the mean conformations from the largest groups (Fig. 5; Supplementary Table S1).

### Monomers of NEU1/TM1 and NEU1/TM2 and dimers of NEU1/TM2 are stable in POPC bilayer

The stability of single NEU1 TM monomers was first examined with the help of full-atom MD simulations in POPC bilayer. For NEU1/TM1, the influence of D145 and E147 protonation state was also tested. It was demonstrated that the negatively charged residues D145 and E147 are not stable in the membrane environment (data not shown). By contrast, for the protonated state, the stable conformation was found with large tilt angle of the helix axis (Supplementary Fig. S4A).

For NEU1/TM2, only N- and C-terminal residues are partially unfolded at the end of the simulation but the main part of the α-helix including region of interest P316-S333 remains intact (Supplementary Fig. S4B). There was also a change in the tilt angle to adopt the hydrophobic length of NEU1/TM2 peptides to the membrane thickness.

The two possible dimer structures of NEU1/TM2 obtained using PREDDIMER (Supplementary Table S1) were also studied in full-atom MD simulations in POPC bilayer. Like the corresponding monomers, these dimers preserve their geometrical parameters and secondary structure during 100 ns MD run. Only N- and C-terminal residues were partially destabilized, while the region P316-S333 remained intact (Supplementary Fig. S4C). Moreover, good preservation of the crossing angle in dimers led us to the assumption that these two conformations may appear in native-like conditions. Noteworthy, for model 2, inter-helical hydrogen bonds were detected. The most stable was H-bond between residues S326 of both helices, and less stable were S333-N334 and N334-H337. To investigate the dimers’ strength, we calculated the free energy of helix association in membrane.

### Free energy of NEU1/TM2 homodimerization in POPC bilayer

**Figure 6:**
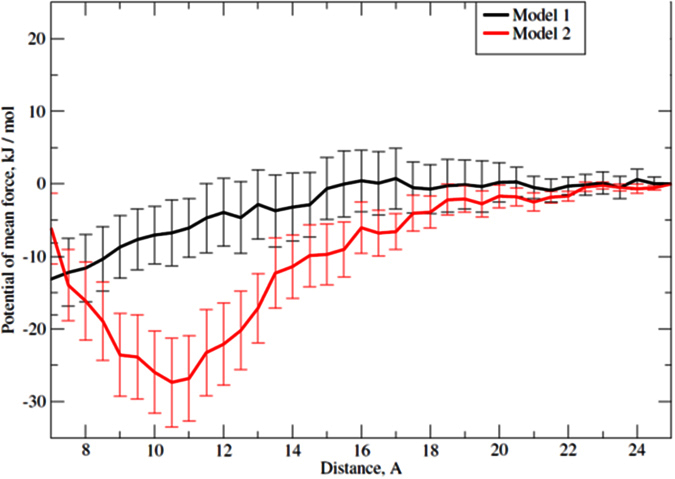
Potential of mean force (PMF) profiles for NEU1/TM2 in POPC bilayer generated using MD simulations with umbrella sampling. Energy profiles for the homodimer of NEU1/TM2, models 1 and 2 are shown in black and red, respectively. Distance between centers of mass of the peptides is taken as a reaction coordinate. Statistical errors are indicated with vertical lines.

The series of MD runs were analyzed in terms of the mean force acting between NEU1/TM2 monomers in POPC bilayer. As the distance between centers of mass was selected as a reaction coordinate, we plotted the force projection on this axis against the distance, and integrated this function. Free energy graphs are shown in Fig. 6. Model 2 reveals much lower energy minimum corresponding to approximately −27 kJ/mol at the distance of 1.1 nm, while model 1 is more tightly packed and has only −13 kJ/mol energy minimum at 0.8 nm distance. We predicted that both interfaces allow favorable dimer formation with different properties and strength, but model 2 seems to be more reliable because it is twice more stable and also allows inter-helical hydrogen bond formation.

### Point mutations in the region 316–333 affect NEU1 dimerization

**Figure 7:**
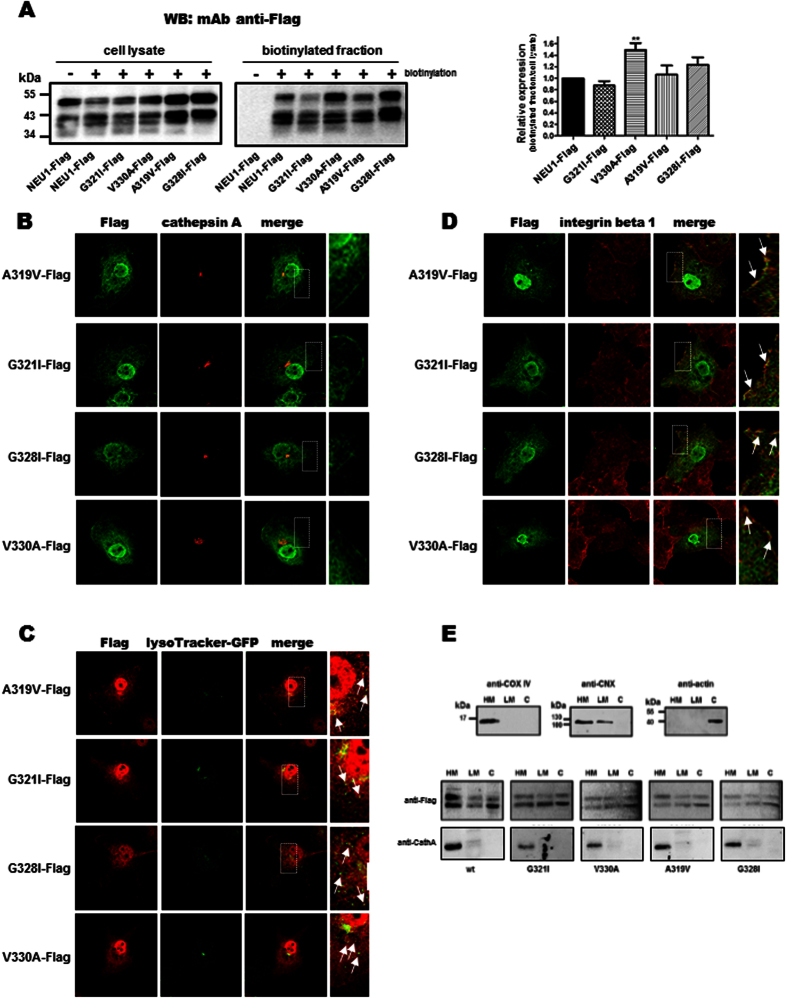
Characterization of the NEU1-Flag mutant expression in COS-7 cells. (**A**) Top, western blot on the biotinylated fraction of COS-7 cells co-transfected with the different NEU1-Flag mutants and PPCA (1:2), and probed with a mouse monoclonal anti-Flag antibody. A representative pattern is shown. Bottom, quantification of the relative expression of the NEU1-Flag mutants in the biotinylated fraction by densitometry analysis. Relative expression was calculated as amount of NEU1-Flag mutant recovered in the biotinylated fraction over expression level in cell lysate and normalized to the control (NEU1-Flag). Results are expressed as mean ± SEM of 4 independent experiments. **P < 0.01. (**B**–**D**) Confocal images of (**B**) NEU1-Flag mutants and cathepsin A, or (**C**) NEU1-Flag mutants and lysosome, or (**D**) NEU1-Flag mutants and integrin beta 1 distribution in permeabilized COS-7 cells co-transfected with NEU1-Flag mutants and PPCA (1:2). (**E**) Subcellular distribution of the different NEU1-Flag mutants. Co-transfected COS-7 cells were lysed and subfractioned in heavy membrane (HM), light membrane (LM) and cytosolic (C) fractions. Equal amounts of proteins were subjected to SDS-PAGE and western blotting. Antibody directed against cytochrome c oxidase (COX IV), calnexin (CNX) and actin were used as markers of the different fractions. A representative pattern of three experiments is shown.

To confirm that the region NEU1/TM2 represents a dimerization interface for NEU1, point mutations were introduced in this sequence. A319V and V330A mutations were performed in order to change the hydrophobicity pattern while potentially inducing few modification of the structure. G321I and G328I mutations were realized in order to provoke more dramatic structural changes via addition of a hydrophobic side chain in a putative region of tight helix-helix packing. The four NEU1-Flag mutants were constructed and first checked for their expression and subcellular localization in COS-7 cells. As shown in Fig. 7A, the different NEU1-Flag mutants were recovered in the plasma membrane fraction as wild-type NEU1-Flag, indicating that each point mutation had minor effects on targeting of NEU1 mutants to the plasma membrane. Densitometric analysis of their relative expression in the plasma membrane fraction was compared to wild-type NEU1-Flag and showed no significant variation except for V330A-Flag mutant. As for wild-type NEU1-Flag (Fig. 2D), immunofluorescence experiments on NEU1-Flag mutants showed both intracellular staining with some co-localization areas with PPCA (Fig. 7B) and lysosomes (Fig. 7C), but also staining at the cell periphery (Fig. 7D) with no evidence for co-localization with PPCA (Fig. 7B). Here again, no staining was observed in non-permeabilized cells suggesting that the C-terminus extremities of the membrane-bound NEU1-Flag mutants are located inside the cells. Subcellular compartmentalization of the different NEU1-Flag mutants was finally investigated by subcellular fractionation using a detergent-free differential centrifugation protocol. The heavy (classically plasma membrane, mitochondria, rough endoplasmic reticulum), light (classically smooth endoplasmic reticulum, free polysomes) and cytosolic fractions were recovered. As expected, COX IV (mitochondria marker) was found in the heavy fraction, calnexin (CXN, endoplasmic reticulum marker) in both heavy and light fractions as previously reported^32^, and actin (cytosol marker) in the cytosolic fraction, thereby confirming fractionation efficiency. When looking at the different NEU1-Flag mutants, their repartition within the different subcellular fractions was comparable to wild-type NEU1-Flag, here again indicating that each point mutation had minor effects on subcellular localization of NEU1 mutants (Fig. 7E). Checking for the presence of PPCA revealed that PPCA was only detected in HM fraction, further demonstrating that NEU1 can be present in some subcellular fractions in the absence of PPCA (Fig. 7E). Taken together, all these results demonstrate that each NEU1-Flag mutant behave as wild-type NEU1-Flag in terms of expression levels, plasma membrane targeting and subcellular localization.

**Figure 8:**
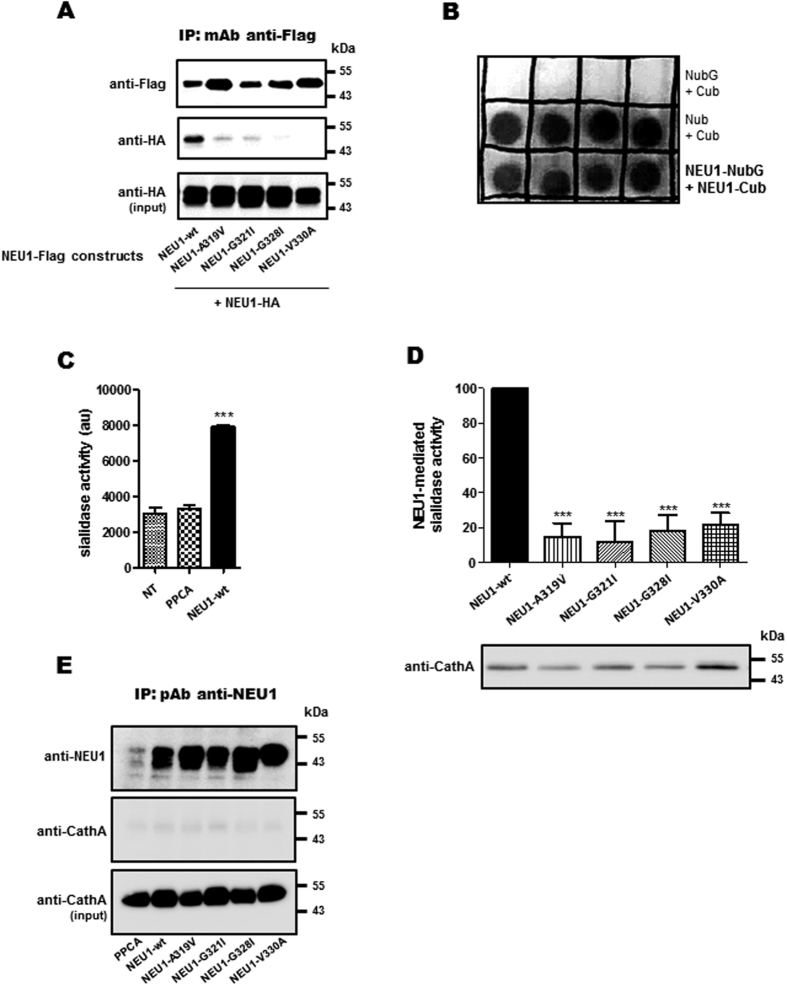
Point mutations in NEU1/TM2 strongly affect NEU1 dimerization and membrane sialidase activity. (**A**) The NEU1-Flag constructs were immunoprecipitated from crude membrane fractions of co-transfected COS-7 cells using a mouse monoclonal anti-Flag antibody and co-immunoprecipitated NEU1-HA was monitored by western blot using a rabbit monoclonal anti-HA antibody. The figure is representative of 3 independent experiments. (**B**) Direct interaction between NEU1 monomer was measured by split-ubiquitin yeast two-hybrid screen. Yeast cells were transformed with NubG and Cub (negative control), Nub and Cub (positive control) or NEU1-NubG and NEU1-Cub constructs. Yeast growth was challenged on minimal growth medium depleted of Trp and Leu by spotting four independent transformants on the different media. (**C**,**D**) Sialidase activity was measured in crude membrane fractions of COS-7 cells co-transfected with the different NEU1 constructs and PPCA (1:2). 50 μg/well of proteins were incubated with 200 μM of 2′-(4-methylumbelliferyl)-alpha-D-N-acetylneuraminic acid substrate for 2 h at 37 °C in MES 20 mM, pH 4.5. (**D**) Sialidase activity was expressed as normalized NEU1-mediated sialidase activity; e.g. sialidase activity mediated by transfected NEU1 (wild type or mutants) over their expression levels revealed by polyclonal anti-NEU1 antibody. Results are expressed as mean ± SEM of 3 independent experiments, each run in duplicate and normalized to NEU1 wt. ***p < 0.001. (**E**) The NEU1 constructs were immunoprecipitated from crude membrane fractions of co-transfected COS-7 cells using a rabbit polyclonal anti-NEU1 antibody and co-immunoprecipitated PPCA was monitored by western blot using a mouse monoclonal anti- cathepsin A.

Co-immunoprecipitation experiments were next performed from crude membrane preparations of transfected COS-7 cells. When NEU1-Flag was co-expressed with NEU1-HA, co-immunoprecipitation of NEU1-HA with wild-type NEU1-Flag was clearly detected (Fig. 8A). However, when co-immunoprecipitations were performed with the NEU1-Flag mutants, the co-immunoprecipitation of NEU1-HA was abolished (Fig. 8A). These results demonstrate that NEU1 can form dimers in membrane and that introducing point mutation in the region 316–333 of NEU1 disrupts dimerization. Moreover, direct interaction between NEU1 monomers was confirmed by split-ubiquitin yeast two hybrids (Fig. 8B).

### Disruption of NEU1 dimerization is associated with decrease in membrane sialidase activity

We finally investigated if disruption of NEU1 dimerization may affect membrane sialidase activity. The latter one was therefore measured from crude membranes of COS-7 cells co-expressing PPCA and the different NEU1 mutants. To avoid any interference with the Flag tag, the mutants were expressed in COS-7 cells as non-tagged constructs. As shown in Fig. 8C, although expression of PPCA alone had no effect on sialidase activity measured in membrane compared to non-transfected cells, co-expression with NEU1 significantly (by 2, 6 times) increased sialidase activity. When the NEU1 mutants were co-expressed with PPCA, a significant decrease (80–85%) in membrane sialidase activity was noted (Fig. 8D). To further confirm that this reduced sialidase activity was not due to variation in PPCA expression and/or modification of NEU1/PPCA interaction in our membrane preparations, the expression level of PPCA was systematically checked and co-immunoprecipitations were performed. No variation in PPCA expression was observed (Fig. 8D) and no interaction between NEU1 constructs and PPCA was detected (Fig. 8E) as reported above (Figs 2A,D and 7B).

## Discussion

The role of NEU1 as a component of the lysosomal complex has been described for decades. NEU1 is localized in lysosomes, where its catalytic activity requires its close association with PPCA and β-galactosidase. In contrast, NEU1 involvement in cellular regulatory mechanisms has been demonstrated only recently concomitantly with the discovery of a plasma membrane-associated pool of NEU1 in several cell types. At the cell surface, NEU1 has been shown to regulate sialylation of several receptors and their underlying signaling pathways^9^,^10^,^12^,^14^,^15^,^16^,^17^,^18^,^19^. However, the mechanisms through which NEU1 might translocate to the plasma membrane, its association with PPCA, its orientation and organization within the membrane are poorly understood and subject to several discrepancies. For instance, the presence of a NEU1-mediated sialidase activity at the cell surface suggests that the active site of NEU1 is extracellular and is conflicting with the acidic optimum pH of the enzyme. In addition, a tyrosine-containing lysosomal targeting motif in the C-terminus of NEU1 (residues 412–415) has been reported in COS-7 cells, human skin fibroblasts and lymphocytes^8^. Upon tyrosine phosphorylation of this motif, NEU1 is redistributed to the cell surface. Taken together, these data suggest that NEU1 C-terminus is intracellular and that NEU1 may contain TM domain(s). However, when looking at the different structural models reported so far for NEU1 that were based on the crystal structure of cytosolic NEU2, no model can account for such orientation.

In this study, we demonstrated that part of NEU1 is indeed recovered at the plasma membrane of COS-7 cells, with both its C- and N-termini most likely lying in the cytosol. Unexpectedly, PPCA was not detected in the biotinylated fraction suggesting that PPCA neither contains extracellular epitopes nor is co-eluted with NEU1 from the plasma membrane fraction. Importantly, these results were obtained from both COS-7 cells overexpressing NEU1 and PPCA and from human macrophages expressing both proteins endogenously, and suggest the existence of an active pool of NEU1 in membrane outside a complex with PPCA, as already reported in erythrocytes^33^. This finding was strengthened by our immunofluorescence and co-immunoprecipitation experiments.

Assuming such a membrane topology, we investigated whether NEU1 may contain TM domains as amino acid sequence analysis of human NEU1 showed that helical regions of hydrophobic nature could be present. Spatial structure and dimerization ability of the peptides containing the predicted most probable TM segments were further studied in direct biophysical experiments using NMR and CD methods. It was found that both peptides penetrate into the hydrophobic core of the membrane-mimicking detergent micelles with different length of the hydrophobic tails and fold into a helical conformation. Some local changes in both TM segments have been however observed depending on the length of the hydrophobic tails. This does not change the overall conclusion that both TM segments fold in a helical conformation in a membrane-like environment but requires further investigations to better understand the influence of the different membrane mimetics on the TM structure. Based on NMR data, it was also concluded that the TM core of the peptides corresponds to regions 139–159 (NEU1/TM1) and 316–333 (NEU1/TM2). Therefore, the predicted boundaries of the former segment were considerably revised as compared with the sequence-based predictions. In addition, the peptide NEU1/TM2 was shown to be capable of dimerization in membrane-mimicking environment, thus indirectly confirming the results obtained with full-size NEU1 (co-immunoprecipitation from crude membrane preparations and direct interaction between NEU1 monomers by split-ubiquitin yeast-two hybrids). The second TM candidate (TM1, region 139–159) has lower potency of dimerization, but can also adopt stable α-helical conformation in membrane environment. Further detailed experimental studies of the dimerization specificity of NEU1 TM segments are required.

Based on the unambiguous structural data, we explored in more detail conformational preferences of the two TM peptides in model membranes *via* computational experiments. MD simulations in explicit lipid bilayers of different nature (“rigid” DPPC and “fluid” POPC) clearly demonstrated stability of the helical conformation of the monomers. Furthermore, we focused on the region 316–333 (TM2) and demonstrated by a combination of independent molecular modeling approaches (massive coarse-grained MD screening from independent starts, systematic MD-search and MD-based free energy calculations) that this domain may self-associate and form homodimers in membrane-like environments (DPPC and POPC).

Although our approach was designed and applied to the peculiar pool of NEU1 present at the plasma membrane, similar organization could also be reasonably envisaged for the lysosomal pool of NEU1. Nevertheless, one should be cautious about the nature and the composition of the lysosomal membrane that can be different from the plasma membrane. TM topology and dimerization of NEU1 have never been reported so far. By studying the interaction between soluble recombinant NEU1 and PPCA, Bonten *et al*. have reported that in the absence of PPCA, NEU1 can self-associate into inactive chain-like oligomers^21^. PPCA presumably competes with NEU1 for the same binding site and can reverse the self-association of NEU1. If these findings apply to this peculiar fraction of NEU1 present at the plasma membrane remains to be shown. Indeed, the majority of NEU1 is intracellular and only a very small fraction is found at the plasma membrane. The absence of any detectable interaction between PPCA and NEU1 all along our study rather suggests that this mechanism of interaction between NEU1 and PPCA may not apply to membrane-bound NEU1. How this pool of NEU1 is targeted to the plasma membrane and how PPCA contributes to this process remain to be further studied. It is tempting to speculate a different mechanism of trafficking, where NEU1 could somehow dissociate from PPCA and move to the membrane or remains associated in the PPCA-NEU1-βGal complex to move to the lysosomes. By introducing point mutations in the region 316–333 of NEU1, we also reported that disruption of NEU1 dimerization was associated with a significant decrease in membrane sialidase activity. Therefore, these results strongly suggest that NEU1 dimerization might be required for catalytic activity of the enzyme within the membrane and identify the region 316–333 as a critical domain involved in this process.

**Figure 9:**
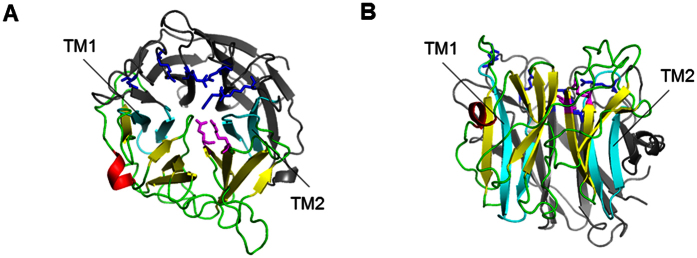
NEU1 structural model and localization of the two potential TM domains. (**A**) Top and (**B**) side view projections of the homology-based NEU1 model. Beta-sheets are shown in yellow, alpha-helices in red and loops in green for the “extracellular” part (between TM domains). Potential TM domains are colored in cyan, and the N- and C-terminal parts are colored in gray. Residues that may form catalytic center (determined by similarity) are shown in magenta for the “extracellular” part and in blue for N- and C-terminal part.

When looking at the structural homology-built model of NEU1 shown in Fig. 9, we agree that the proposed TM domains seem not compatible with such model and the well-known β-propeller structure. However, as mentioned previously, all the NEU1 models reported so far are built from the crystal structure of the human cytosolic NEU2. Importantly, among human sialidases, the overall amino acid identity of NEU1 to the other sialidases is relatively low (19–24%) whereas NEU2, NEU3, and NEU4 show 34–40% homology to each other^4^. The two regions proposed as transmembrane α-helices (TM1 and TM2) are found either in β-sheets or in loops, according to the β-propeller structure accepted so far. Therefore, this organization would result into the catalytic residues being separated by the lipid bilayer: R78 (a member of the arginine triad), D103 (involved in substrate recognition and catalysis) and Y370 (involved in catalysis) would be on the cytosolic side, whereas R280, R341 (the other members of the arginine triad) and E264 (involved in substrate recognition and catalysis) would be outside the membrane.

Importantly, beta-propeller folds have remarkable structural plasticity and are prone to strand-swapping between blades, large insertions of entire functional beta-sheet domains, thus making possible assembling of functional supramolecular beta-propeller units^34^,^35^. Therefore, one can speculate that the dissecting of the usual beta-propeller fold (as shown in Fig. 9) by introducing two TM segments is compensated by dimerization of NEU1 both in extracellular and cytoplasmic parts. Indeed, the corresponding sequences (between TM1/TM2 and N/C-terminal regions, respectively) reveal high propensity to form beta-structural blades – this follows from the results given by fold recognition techniques (not shown). In NEU1 dimers, the sizes of these two parts become sufficient to adopt a proper full-size beta-propeller fold on the both sides of the plasma membrane. Obviously, this is just a hypothesis, which tries to reconcile the common view on structural organization of sialidases and our new experimental data about TM topology of NEU1. Further works are required to confirm this hypothesis.

In conclusion, this study raises important questions about our current understanding of NEU1. As underlined by Giacopuzzi *et al*.^2^, NEU1 has several sequence specificities, notably in the region 316–333, which make it a unique enzyme in the sialidase family. Consequently, alternative folding could be considered even though the NEU1 conformation would be most probably not very different from a strict six-bladed beta propeller. Finally, we shall now reconsider the current molecular models obtained for NEU1 in keeping with the likely presence of this dimerization TM domain (TM2) and envisage that a second one (TM1), is also present.

## Materials and Methods

### Plasmid constructs

cDNA encoding human PPCA was kindly provided by Pr Alessandra d’Azzo and subcloned in pcDNA_3_ vector. NEU1 cDNA was from ImaGenes GmbH (Berlin, Germany). The NEU1-Flag construct was obtained using NEU1 cDNA as template and the Phusion High-Fidelity DNA Polymerase (ThermoScientific). Restriction sites for HindIII and BamHI were introduced by PCR. After digestion by the respective restriction enzymes, the resulting insert (NEU1) was ligated into the p3XFlag-CMV^TM^-14 vector (Sigma) encoding three adjacent Flag epitopes at the C-terminus of the fusion protein. The different NEU1-Flag and NEU1 mutant constructs were obtained by PCR-based site-directed mutagenesis. Internal primers were used to generate the A319V, G321I, G328I, G328S and V330A mutations. The HA-NEU1 and HA-NEU1-Flag constructs was obtained by introduction of a 2xHA tag after the predicted signal peptide (AA 1–47)^23^ of NEU1 using the QuickChange II site-directed mutagenesis kit (Agilent Technologies). All cDNA sequences were confirmed by sequencing.

### Antibodies

Mouse monoclonal anti-Flag M2, rabbit polyclonal anti-Flag and anti-calnexin antibodies were from Sigma. Rabbit monoclonal anti-HA antibody was purchased from Cell Signaling and rabbit polyclonal anti-NEU1 (H-300), mouse monoclonal anti-cathepsin A, and goat polyclonal anti-actin antibodies from Santa Cruz. Mouse monoclonal anti-COX IV antibody was from Abcam and purified rat anti-human CD29 (integrin beta 1) from BD Biosciences. Dylight 488 conjugated-mouse monoclonal anti-HA antibody was purchased from ThermoFisher Scientific and Alexa Fluor 488 or 568-conjugated donkey anti-mouse, rat or rabbit antibodies from Invitrogen.

### Cell culture and transfections

COS-7 cells were cultured in Dulbecco’s modified Eagle’s medium supplemented with 10%(v/v) fetal bovine serum, 100 units/mL penicillin, 0.1 mg/mL streptomycin at 37 °C in a humidified atmosphere at 95% air and 5% CO_2_. Transient transfections were performed with JET-PEI (Polyplus Transfection), according to the manufacturer’s protocol.

### Biotinylation experiments

COS-7 cells grown in 10 cm plates were co-transfected with 2 μg of the different NEU1 cDNA constructs together with 4 μg of PPCA cDNA for 48 hours. Alternatively, human macrophages differentiated from monocytes by human M-CSF (100 ng/mL, 1 week, Preprotech) were used. Adherent cells were washed three times in PBS, incubated with 0.5 mg/mL of EZ-Link^®^ sulfo-NHS-LC-biotin (ThermoScientific) for 30 min at 4 °C, and quenched with 100 mM glycine (30 min, 4 °C). The cells were then scraped in TEM buffer (75 mM Tris, 2 mM EDTA, 12 mM MgCl_2_, protease inhibitor cocktail, 10 mM NaF, 2 mM Na_3_VO_4_, pH 7.5) containing 1% Triton X-100, sonicated and incubated under gentle end-over-end mixing (3 h, 4 °C) for solubilization of membrane proteins. Lysates were centrifuged (20,000 g, 45 min, 4 °C) to pellet insoluble material and supernatants incubated with 20 μL streptavidin agarose beads (GE Healthcare) for 45 min at 4 °C to purify biotinylated membrane proteins. After several washes, biotinylated proteins were eluted from the beads by Leammli buffer and subjected to SDS-PAGE and immunoblotting. Immunoblottings were performed using the mouse monoclonal anti-Flag antibody (1/1000), mouse monoclonal anti-cathepsin A (1/1000), or rabbit polyclonal anti-NEU1 (1/1000) and immunoreactivity was revealed using a HRP-conjugated anti-mouse or -rabbit antibody (1/10,000) followed by enhanced chemiluminescence detection reagents (GE Healthcare) and visualized with the Odyssey Fc LI-COR scanner (ScienceTec).

### Immunofluorescence

COS-7 cells transiently transfected by 0.25 μg of the different NEU1-Flag cDNA constructs or HA-NEU1-Flag cDNA together with 0.5 μg of PPCA cDNA were grown on sterile coverslips in 24-well plates. 48 post-transfection, cells were washed three times in PBS, fixed with 2% paraformaldehyde in PBS for 15 min and permeabilized, or not, by 0.2% Triton X-100 in PBS for 10 min. After blocking with 3% BSA in PBS for 1 h, cells were incubated with rabbit polyclonal anti-Flag (2 μg/mL) and mouse monoclonal anti-cathepsin A (1 μg/mL) or purified rat anti-CD29 (2 μg/mL) in PBS containing 0.3% BSA for 1 h at room temperature. Coverslips were then washed three times with PBS and incubated with Alexa Fluor 488-conjugated donkey anti-rabbit and Alexa Fluor 568-conjugated donkey anti-mouse or rat antibodies (1:1000) in PBS containing 0.3% BSA for 1 h at room temperature. For colocalisation studies between NEU1-Flag and lysosomes, adherent cells were incubated for 16 h at 37 °C with a lysoTracker (CellLight^®^ lysosomes-GFP, Molecular Probes). Then, cells were fixed and permeabilized as above and incubated with a monoclonal anti-Flag M2 (2 μg/mL) and Alexa Fluor 568-conjugated donkey anti-mouse antibodies (1:1000). Coverslips were mounted, visualized with a laser scanning microscope (LSM 710 NLO, Zeiss) and analyzed by Image J software.

### Flow cytometry

COS-7 cells grown in 10 cm plates were co-transfected with 2 μg HA-NEU1, NEU1-HA or empty vector cDNA together with 4 μg PPCA cDNA. Forty-eight hours after transfection, cells were washed two times in PBS and recovered after incubation with 2 mM EDTA (10 min, 37 °C). Cells were then adjusted to 1 million/tube and subjected to labelling following prior permeabilization, or not, by 0.1% saponin. The Dylight 488 conjugated-mouse monoclonal anti-HA antibody was used at 1:50 and incubated for 30 min at 4 °C. After washes, cells were resuspended in PBS containing 1 mM EDTA and 1% PFA and analyzed with a LSR Fortessa flow cytometer (BD Biosciences). Acquisition and processing data from 50,000 cells were performed and analyzed using the BD FACSDIVA software (BD Biosciences). Cell population was gated using their forward and side scatter characteristics. Positive labelling was determined by comparing the fluorescence of HA-NEU1 and NEU1-HA expressing cells versus empty vector expressing cells.

### Subcellular fractionation

COS-7 cells grown in 10 cm plates were co-transfected with 2 μg of the different NEU1-Flag cDNA constructs together with 4 μg of PPCA cDNA. Forty-eight hours after transfection, cells were washed twice with PBS and lysed in fractionation buffer (20 mM HEPES, 250 mM sucrose, 10 mM KCl, 1.5 mM MgCl_2_, 1 mM EDTA, 1 mM EGTA, 1 mM DTT, protease inhibitor cocktail, 10 mM NaF, 2 mM Na_3_VO_4_, pH 7.4). Cell lysate was then passed through a 25 G needle 10 times and centrifuged at 800 g for 5 min at 4 °C to remove nuclei and unbroken cells. The post-nuclear supernatant was centrifuged at 10,000 g (15 min, 4 °C) to collect the heavy membrane (HM) fraction (pellet), and then at 100,000 g for 1 h to obtain the light membrane (LM) fraction (pellet) and the cytosol (supernatant). Both HM and LM fractions were washed with fractionation buffer, resuspended by pipetting, passed through a 25 G needle 10 times, and centrifuged again as above. After centrifugation, the pellets were resuspended in Laemlli buffer and the HM, LM and cytosol fractions were subjected to western blotting for detection of PPCA and the NEU1-Flag constructs. The endogenous markers used were: COX IV (as a mitochondrial protein), calnexin (as an ER protein), and actin (as a cytosolic protein).

### Nuclear Magnetic Resonance (NMR) and Circular Dichroism (CD) studies of NEU1 transmembrane fragments in membrane-like environment

#### Bacterial expression and purification of the hNEU1/TMS2 fragment corresponding to the NEU1 residues R^305^-R^341^

The plasmid construct is based on the *pGEMEX-1* vector (Promega). The fragment of human NEU1 gene, corresponding to the residues R^305^-R^341^ (named as hNEU1/TMS2), was amplified by PCR with flanking primers. Upstream to the hNEU1/TMS2 gene in the *pGEMEX/TRX-hNEU1/TMS2* vector thrombin site was inserted. The thioredoxin (*TRX*) gene with downstream protease site and *hNEU1* gene with thrombin insertion were recombinated. The recombinant product was hydrolyzed by *RsrII* and *BamHI* restrictases (the sites are located near 5′-end of the *TRX* gene and 3′-end of the *hNeu1tm* gene, correspondingly) and then ligated with the vector preliminary subjected to the hydrolysis by the same restrictases. DNA of the selected clones was sequenced within the insertion. *E. coli Rosetta*(*DE3*)*/pLysS* cells were transformed by the *pGEMEX/TRX- hNeu1tm* plasmid and then plated into dishes with ampicillin and chloramphenicol in concentrations 35 and 100 mg/mL, respectively, and then incubated at 37°С overnight. 250 colonies from the plate were inoculated into 250 mL of auto-induction medium C-750501 (0.75% glycerol, 0.05% glucose, 0.01% lactose)^36^, containing ^15^NH_4_Cl (CIL, USA) for the production of the ^15^N-labeled peptide, and incubated at 25 °C at 300 rpm on orbital shaker for 72 h. 250 mL of culture media were centrifuged and lyzed in 30 mL of buffer A containing 50 mМ Tris-HCl pH 8, 4 М urea, 0.25 М NaCl, 1% Triton X–100, 0.1 mМ phenylmethylsulfonylfluoride. Suspension was sonicated 10 times for 30 s of 60 W pulses on ice bath and centrifuged at 15,000 g for 20 min. The supernatant was applied on Ni-sepharose column preliminary equilibrated with buffer A. Column was washed with buffer B (the same as buffer A but without urea and PMSF) for removing urea and buffer C (the same as buffer B but with 20 mM imidazole) for removing nonspecifically bound proteins. Target protein was eluted with buffer E (the same as buffer B but with 200 mM imidazole). The eluted fractions were analyzed by tris-glycine electrophoresis in 12% gel. The eluate was diluted 5-fold with buffer D (50 mM Tris-HCl pH 8.0, 0.5% Triton X-100) for subsequent proteolysis and subtractive chromatography. Hydrolysis of the hybrid protein was carried out at room temperature; 5 NIH activity units of thrombin were added and mixture was incubated for 16 h. After that solution was centrifuged at 15,000 g for 20 min. The supernatant was applied on Ni-sepharose column preliminary equilibrated with buffer D (20 mM Tris-HCl pH 8, 1% Triton X-100). The target protein, not bound to the column, was concentrated 3 times by vacuum drying, precipitated by addition of 1/10 (v/v) of trichoracetic acid (TCA). After 15 min incubation at −20 °С tubes were centrifuged at 12,000 g for 15 min. Then, precipitate was washed by acetone 3 times (with 15 min incubation at −20 °С between centrifugations) to remove detergent. The precipitate was dissolved in 1/1 (v/v) mixture of trifluoroethanol/water and lyophilized for storage or incorporation into membrane-mimetic milieu. Peptide purity was verified by SDS-page and by analyzing the ^1^H/^15^N-HSQC NMR spectrum of the ^15^N-labeled peptide.

#### Cell-free expression and purification of the hNEU1/TMS1 fragment corresponding to the NEU1 residues D^130^-S^180^

Plasmid constructs were obtained based on pGEMEX-1 expression vector (Promega). The fragment of human the *hNeu1* gene, corresponding to the residues D^130^-S^180^ with the amino acid substitution Cys164Ser (named as hNEU1/TMS1), was amplified by PCR from overlapping primers with restriction sites at the 5′- ad 3′-ends. PCR fragments were hydrolyzed by the restriction endonucleases NdeI and HindIII, and ligated with the vectors, treated with the same restrictases. Clones with the required insertions were selected by the PCR and sequenced within the insert. Plasmid construct expressed in continuous exchange cell-free (CECF) system with bacterial S30-extract and T7-polymerase which were obtained from *E. coli* Rosetta(DE3)PLysS strain (Novagen) according to the protocol^37^. The reaction was performed in 50-mL tubes using the 12.4 kDa dialysis membrane, closed from the both ends by the dialysis clips. The reaction mixture (RM)/feeding mixture (FM) ratio was 1/6. The reaction components incubated at 150 rpm in the Innova 44 R shaker at 34 °C during 16–20 h. The final concentrations of the components in RM were: 100 mM HEPES-KOH pH 8, 15 mM Mg(OAc)_2_, 80 mM KOAc, 20 mM potassium acetylphosphate, 60 mM sodium creatine phosphate, 0.24% mass fraction of the total ^13^C^15^N-labeled algal amino acids (or 1 mM equimolar mix of the unlabeled amino acids), 0.1 mg/mL folinic acid, 1.2 mM ATP and 0.8 mM GTP, CTP and UTP, protease inhibitors without EDTA, 0.5% NaN_3_, 2 mM dithiothreitol, 2% polyethylene glycol with molecular mass 8 kDa, 0.3 unit/μL ribonuclease inhibitor, 0.12 mg/mL creatine kinase, 50 μg/μL plasmid DNA, 0.5 mg/mL total *E. coli* MRE600 RNA, ^15^N-labeled mix of 20 amino acids, 35% of the total volume S30 *E. coli* extract. The precipitate after the translation process was collected; the dialysis membrane was washed by 2 mL of the ultrapure water and centrifuged at 18 000 g during 15 min at 23 °C. The reaction mixture precipitate was washed by the buffer contained 10 μg/mL RNAse A, 20 mM Tris-HCl pH 8, 100 mM NaCl, and incubated at 30 °C with stirring during 30 min. Then it was centrifuged at 18 000 g during 20 min at 23 °C and solubilized in buffer contained 1% sodium lauroyl sarcosyl, 50 mM Tris-HCl pH 8, 50 mM NaCl and 1 mM EDTA. Chromatography buffer contained the same components except for EDTA. Size-exclusion chromatography run on the Tricorn 10/300 column filled with the Superdex 200 prep grade sorbent at the flow rate 0.5 mL/min. 0.8 mL fractions were collected and analyzed using 12% PAAG-electrophoresis in the Tris-tricine buffer system. After the chromatography, fractions containing the target protein were pooled and the protein was precipitated using trichloroacetic acid at the 1/10 vol ratio with the subsequent 3-times acetone washing. The precipitate was solubilized in the trifluoroethanol/water mix 1/1 (v/v) and used for incorporation into the micelles or lyophilized for a storage. Peptide purity was verified by SDS-page and by analyzing the ^1^H/^15^N-HSQC NMR spectrum of the ^15^N-labeled peptide.

#### Solubilization of the hNEU1/TMS2 and hNEU1/TMS1 fragments in membrane-mimicking micellar environment

The detergent micelles consisted of Fos-Choline-12 (having the acyl chain length of 12), usually called n-dodecylphosphocholine (DPС), and Fos-Choline-16 were used as a membrane-mimicking environment. The ^15^N-labeled hNEU1/TMS2 and hNEU1/TMS1 fragments were incorporated into the detergent micelles with an effective detergent/protein molar ratio (D/P) varied from 50 to 200 at total detergent concentrations varied from 80 to 21 mM, respectively. The peptide’s powder was first dissolved in 1/1 (v/v) trifluoroethanol-water mixture with addition of the detergents, then kept for several minutes in an ultrasound bath and lyophilized. After that, the dried ^15^N-labeled samples were dissolved at pH 6.6 in 400 μL of 20 mM phosphate buffer solution, containing 0.15 μM sodium azide, 1 mM EDTA, and 5% D_2_O. In order to prevent intermolecular SS-bonding of C^158^, 2 mM of methyl ester derivative of TCEP (tris-2-carboxyethyl phosphine) was added to the hNEU1/TMS1 sample. In order to ensure uniformity of the micelle size, several freeze–thaw cycles were carried out, followed by sonication until the sample became transparent. The 0.5 mM ^15^N-labeled samples of hNEU1/TMS2 and hNEU1/TMS1 in the monomeric (mainly) state was obtained at D/P of 200 (the typical micelle size is 60–80 molecules of DPC). In order to study the peptide dimerization in the membrane-mimicking environment, the titration of the ^15^N-labeled hNEU1/TMS2 sample having initial D/P of 50 at 21 mM total concentration of DPC was carried out by adding of small portions of concentrated micelle suspension up to final D/P of 110 with preservation of the protein concentration near 0.4 mM. Several freeze/thaw cycles were made for the sample at each D/P point. For lower D/P values, the dimer population of hNEU1/TMS2 is increased rapidly but precipitation of the peptide occurs within several hours.

#### CD spectroscopy

CD spectra of the NMR sample of the monomeric hNEU1/TMS2 and hNEU1/TMS1 fragments in DPC and Fos-Choline-16 micelles (at D/P of 200) were measured on spectropolarimeter J-810 (Jasco, Japan) in 0.1 mm quartz cuvette at 20 °С. The contribution of the empty detergent micelles was subtracted. The analysis of the CD spectra was performed with the CONTILL program^38^. The deconvolution of the CD spectrum of hNEU1/TMS2 embedded into DPC micelles revealed the presence of 50.4% of α-helix structure and 7.5%, 16.7% and 25.4% of β-sheet, turn and coil structure, respectively, with NRMSD 0.04%. The deconvolution of the CD spectrum of hNEU1/TMS1 embedded into DPC micelles revealed the presence of 45.8% of α-helix structure and 9.8%, 16.4% and 27.9% of β-sheet, turn and coil structure, respectively, with NRMSD 0.02%. Similar secondary structure distributions were observed in the case of monomeric hNEU1/TMS2 and hNEU1/TMS1 incorporated into Fos-Choline-16 micelles (Supplementary Fig. S1).

#### NMR spectroscopy

The ^1^H/^15^N-HSQC, ^1^H/^15^N-TROSY, ^1^H/^15^N-TOCSY-HSQC (with mixing time of 40 ms) and ^1^H/^15^N-NOESY-HSQC (with mixing time of 100 ms) NMR spectra^39^ were acquired for an identification of the conformation of ^15^N-labeled hNEU1/TMS2 and hNEU1/TMS1 in the membrane-mimicking environment at 40 °C on 800 MHz AVANCE III spectrometer (Bruker BioSpin) equipped with the pulsed-field gradient triple-resonance cryoprobe. Exchange rates between water and HN protons were analyzed by detection of cross-peaks in CLEANEX spectrum^40^, and observed cross-peaks were treated as an indication of water exposed amide groups.

### Molecular modeling of NEU1/TM dimers

#### Probing of self-assembling of NEU1/TM2 dimer via coarse-grained MD simulations in POPC

The starting full-atom ideal α-helical conformation of 32-residue long peptide D310-R341 was constructed in PyMOL. Then, it was converted to coarse-grained representation using Martinize tool and Martini force field version 2.2 P^41^. Four flanking residues on both ends have been assigned to be coiled, while other residues were marked as α-helix. The structure was copied and displaced in XY plane by the distance of 2.5 nm (between the axes). Hydrated bilayer containing 188 POPC molecules, 1780 polarizable water particles and 6 sodium ions was generated using INSANE program^42^. Then, a set of 144 starting conformations was generated by rotating each helix around its axis with 30 degrees’ step followed by energy minimization and relaxation. MD simulations were carried out using GROMACS^42^ version 4.5. Simulation protocol was similar to that used elsewhere with Martini 2.2 P force field^43^. Integration step was 20 fs, each trajectory was 1 μs long. Temperature of 310 K was controlled by V-rescale thermostat algorithm. Berendsen barostat with semi-isotropic pressure of 1 atm was used. Coulombic and van der Waals interactions were shifted at 1.2 nm.

#### Probing NEU1/TM2 dimer via MD simulations in DPPC

To ensure native-like anchoring on the polar lipid-water interface, the potential TM2 peptide ^316^PVVAAGAVVTSSGIVFFS^333^ was extended with seven residues on both termini. MD simulations were thus performed on the 32-residue peptide ^309^FDPELVDPVVAAGAVVTSSGIVFFSNPAHPEF^340^ generated with the ribosome program (http://folding.chemistry.msstate.edu/~raj/Manuals/ribosome.html). The central 19-residue fragment was constructed in α-helical conformation while the termini were built in a geometry shown in Supplementary Fig. S3A. The peptide was replicated, rotated around its main axis and translated in order to get two peptides with parallel axes (Supplementary Fig. S3B). The distance between the axes was equal to 2.0 nm. Twenty-four rotation angles were considered from 0° to 330°. Bilayer of 128 dipalmitoylphosphatidylcholine (DPPC) molecules was downloaded from the Tieleman Website (http://wcm.ucalgary.ca/tieleman/downloads) and the peptides were inserted using the InflateGRO script^44^. MD simulations were conducted using the GROMACS simulation package^45^ and GROMOS53A6 force field^46^. The two peptides were placed in boxes with side length varying from of 6.0 nm to 9.0 nm. Water (SPC model)^47^ and counter ions were added prior to the simulations. In order to relax the structures, 5000 steps of energy minimization were performed using the steepest descent algorithm. The systems were equilibrated for 5 ns at the temperature of 322 K in the isothermal-isobaric ensemble. The equilibration steps were followed by MD simulations carried out for 250 ns, maintaining a pressure of 1 bar (Berendsen algorithm) and a temperature of 322 K (V-rescale algorithm). Time step of 2 fs and the LINCS algorithm were employed^48^. Non-bonded interactions were calculated using the Particle Mesh Ewald (PME) algorithm with a cut-off at 1.2 nm for the Coulombic and van-der-Waals interactions^49^,^50^.

#### Sequence-based prediction of homodimer models for NEU1/TM2

Possible models of NEU1/TM2 (316–336) homodimers were generated using the web-server PREDDIMER^31^ freely available at http://model.nmr.ru/preddimer. To account for independent starting conditions and to assess the effect of the peptide termini, three sequence fragments were used for the calculation. To eliminate structural redundancy in the resulting set of models, cluster analysis was performed. Similarity between the conformers was assessed in terms of root-mean-square deviation (RMSD) between Ca atoms of the peptides, geometrical parameters of a dimer, interface of dimerization and hydrophobic properties distribution on the surface of a dimer^51^. The models with pairwise RMSDs calculated for Cα atoms within 0.2 nm were considered as identical.

#### Probing stability of NEU1/TM1 and NEU1/TM2 monomers and NEU1/TM2 dimer via MD in POPC

Models of NEU1/TM1 and NEU1/TM2 monomers (residues D135-Q165 and D310-R341, respectively) were built in ideal α-helical conformation using PyMOL program. For NEU1/TM1 two protonation states of D145 and E147 were selected. In the first model, these residues were taken in a charged form, and in the second one they were protonated and so uncharged. The two best models of NEU1/TM2 dimers generated by PREDDIMER were modified by addition of flanking residues at the N- and C-termini. These fragments were built as continuation of ideal α-helices to get the 32-residue long peptides: D310-R341 including TM region of interest. After that, the monomers and the dimer were inserted into pre-equilibrated hydrated palmitoyloleoylphosphatidylcholine (POPC) bilayer and the overlapping lipid and water molecules were removed. To get the system electrically neutral, sodium ions were added by replacement of some waters. This procedure resulted in a system comprised of one or two peptides, 220–240 POPC molecules (depending on the selected peptide and conformation), about 16000 water molecules (TIP3P model)^52^ and counterions. AMBER99SB-ILDN and SLipids forcefields^53^,^54^ were used in all MD simulations in the POPC membrane. 10000 iterations of steepest descent energy minimization were performed before the MD simulation followed by relaxation of bilayer with increasing temperature (from 5 K to 315 K) and constrained positions of protein atoms (5 ns long). Electrostatic interactions were treated with the Particle-Mesh Ewald (PME) summation method with 1.0 nm cut-off. Van-der-Waals interactions were truncated at the distance 1.4 nm. V-rescale thermostat with the reference temperature of 315 K was used, and the semi-isotropic pressure coupling was implemented using the Parinello-Rahman barostat^55^. Two independent 100 ns MD trajectories were recorded for each system, and the mean structures of dimers were retrieved for the analysis. The stability of peptides was tested in terms of RMSD, preservation of geometrical parameters and secondary structure. Hydrogen bonds formation was also analyzed for dimers.

#### Free energy of helix-helix association in NEU1/TM2 homodimers

The free energy of dimerization was calculated using the potential of the mean force approach with umbrella sampling^56^. The simulation parameters were the same as on the previous step. In total, 37 windows were generated for each system with different distance (from 0.7 to 2.5 nm) between centers of masses of the monomers. In each window, 10 ns MD-relaxation was done, followed by a 50 ns production run, in which relative positions of the monomers were constrained to a given distance using a harmonic potential. Finally, the mean force acting on the monomers was calculated from the MD data and integrated to get the free energy profile of association. Other details of the simulations can be found elsewhere^57^.

#### Co-immunoprecipitations

Co-transfected COS-7 cells grown in 10 cm plates were washed two times in PBS, resuspended in 1 mL cold TEM buffer (75 mM Tris, 2 mM EDTA, 12 mM MgCl_2_, protease inhibitor cocktail, 10 mM NaF, 2 mM Na_3_VO_4_. pH 7.5). After sonication, crude membranes were pelleted by centrifugation at 20,000 g during 45 min at 4 °C and solubilized during 3 h at 4 °C under gentle end-over-end mixing in 500 μL TEM buffer containing 1% CHAPS. Samples were then centrifuged at 20,000 g during 45 min at 4 °C and the supernatant (solubilized crude membrane proteins) was recovered. Immunoprecipitations were performed using 4 μg of mouse monoclonal anti-Flag or rabbit polyclonal anti-NEU1 pre-adsorbed on protein G sepharose beads (GE Healthcare) for 2 h at 4 °C. Immunoprecipitated proteins were eluted with Laemmli buffer and subjected to SDS–PAGE and immunoblotting. Immunoblottings were performed using the indicated antibodies and immunoreactivity was revealed using a HRP-conjugated secondary antibodies (1/10,000) followed by enhanced chemiluminescence detection reagents and visualized with the Odyssey Fc LI-COR scanner.

### Split-ubiquitin yeast two hybrid

The NEU1 cDNA was subcloned into pDONR221 before integration in the Split-Ubiquitin destination vectors. The Split-Ubiquitin vectors, pMetYC-DEST and pNX35-DEST, were used to produce the Met-repressible bait construct NEU1-Cub-PLV and prey construct NEU1-NubG, respectively. NubWT fragment, pMetYC-DEST and pNX35-DEST vectors were kindly provided by Dr F. Chaumont (Institut des Sciences de la Vie, Université Catholique de Louvain, Belgium). Electroporation-competent THY.AP4 yeasts were co-transformed as described previously^58^ with the Nub and Cub constructs of interest. Yeast colonies co-expressing the bait and prey constructs were recovered 48 h after transfer on vector selective media (CSM, −Leu^−^, Trp^−^) and used to inoculate liquid vector-selective media. The next day, growth assays were performed as followed: yeasts co-expressing the Met-repressible bait construct NEU1-Cub-PLV and the prey constructs NEU1-NubG, NubG (negative control) or NubWT (positive control) were dropped in serial dilutions (0.5, 0.05 and 0.005 in water) onto plates on interaction-selective media (CSM −Leu^−^, Trp^−^, Ade^−^, His^−^) with, or not, addition of 5, 50 and 500 μM Met to repress expression of the bait. Growth was monitored after 48 h at 30 °C.

### Sialidase activity

Co-transfected COS-7 cells grown in 10 cm plates were washed two times in PBS and resuspended in 1 mL cold TEM buffer (75 mM Tris, 2 mM EDTA, 12 mM MgCl_2_, protease inhibitor cocktail, 10 mM NaF, 2 mM Na_3_VO_4_. pH 7.5). After sonication, crude membranes were pelleted by centrifugation at 20,000 g during 45 min at 4 °C and resuspended in 20 mM MES, pH4.5. Sialidase activity was assessed using the 2′-(4-methylumbelliferyl)-alpha-D-N-acetylneuraminic acid (BioSynth) as substrate. Assays were performed in triplicate (each run in duplicate) with 50 μg of crude membranes proteins and 200 μM of substrate for 2 h at 37 °C. Reactions were stopped by adding 1 M Na_2_CO_3_ and the fluorescence of each well was measured in triplicate using an Infinite F200 PRO microplate reader (TECAN). To assess the functionality of the NEU1-Flag, NEU1-HA, NEU1 and HA-NEU1 constructs (Fig. 2F), sialidase activity was directly measured in 12-well plates on adherent cells in the same conditions as described above except that the 2-O-(p-nitrophenyl)-α-D-N-acetylneuraminic acid (Sigma) was used as substrate.

### NEU1 homology model generation

Prediction of the human NEU1 3D structure was done by homology modeling using the SWISS-MODEL software^59^,^60^,^61^ (accessible via the ExPASy web server) and the human NEU2 crystal structure (PDB 2F11) as template.

### Statistical analysis

Results are expressed as mean +/− SEM. Statistical significance was evaluated using one-way ANOVA followed by Dunnett’s multiple comparison test. *P* values of less than 0.05 were considered as statistically significant.

## Additional Information

**How to cite this article**: Maurice, P. *et al*. New Insights into Molecular Organization of Human Neuraminidase-1: Transmembrane Topology and Dimerization Ability. *Sci. Rep.* **6**, 38363; doi: 10.1038/srep38363 (2016).

**Publisher's note:** Springer Nature remains neutral with regard to jurisdictional claims in published maps and institutional affiliations.

## Supplementary Material

Supplementary Information
