# Supplementary material for: New Insights into Molecular Organization of Human Neuraminidase-1: Transmembrane Topology and Dimerization Ability
Source: Sci Rep. 2016 Dec 5;6:38363. doi: 10.1038/srep38363 (PMC5137157; doi:10.1038/srep38363)
Supplement: Supplementary Information [file srep38363-s1.pdf]

SUPPLEMENTARY INFORMATION

NEW INSIGHTS INTO MOLECULAR ORGANIZATION OF HUMAN NEURAMINIDASE-1: TRANSMEMBRANE TOPOLOGY AND DIMERIZATION ABILITY

Pascal Maurice[1,*], Stéphanie Baud[1,2], Olga V Bocharova[3], Eduard V Bocharov[3], Andrey S Kuznetsov[3], Charlotte Kawecki[1], Olivier Bocquet[1], Béatrice Romier-Crouzet[1], Laetitia Gorisse[1,ϕ], Maxime Ghirardi[1], Laurent Duca[1], Sébastien Blaise[1], Laurent Martiny[1], Manuel Dauchez[1,2], Roman G Efremov[3,4,†], Laurent Debelle[1,†].

[1] Laboratoire Signalisation et Récepteurs Matriciels (SiRMa), UMR CNRS 7369 Matrice Extracellulaire et Dynamique Cellulaire (MEDyC), Université de Reims Champagne Ardenne (URCA), UFR Sciences Exactes et Naturelles, Reims, France.
[2] Plateau de Modélisation Moléculaire Multi-échelle, UFR Sciences Exactes et Naturelles, URCA, Reims, France.
[3] M. M. Shemyakin and Yu. A. Ovchinnikov Institute of Bioorganic Chemistry, Russian Academy of Sciences, Moscow, Russia.
[4] Higher School of Economics, Myasnitskaya ul. 20, 101000 Moscow, Russia.

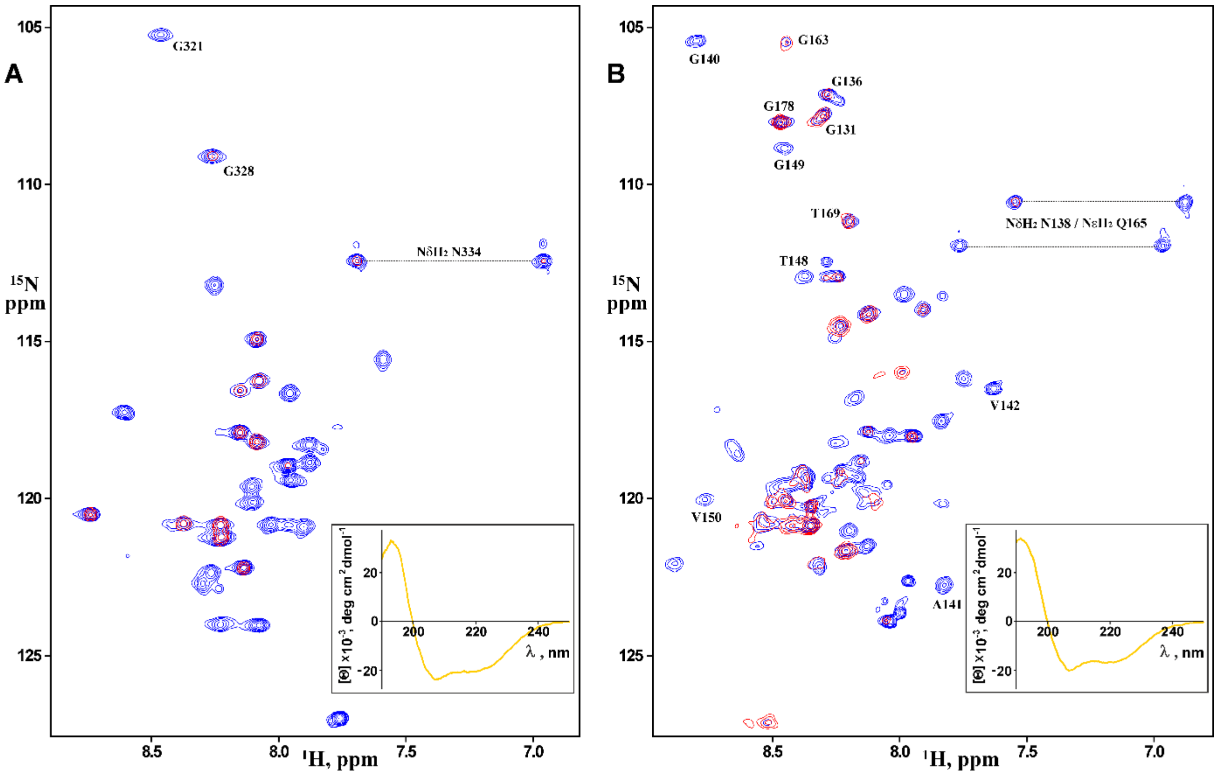

Supplementary Fig. S1 NMR and CD spectra of hNEU1/TMS2 and hNEU1/TMS1 fragments in detergent Fos-Choline-16 micelles. (**A**, **B**) Overlaid NMR spectra in blue and red (the $^{1}H/^{15}N$-HSQC and CLEANEX NMR spectra, respectively) of (**A**) NEU1/TMS2 and (**B**) NEU1/TMS1 fragments embedded into Fos-Choline-16 micelles at the detergent/protein molar ratio (D/P) of 200. Amide cross-peak dispersion in the $^{1}H/^{15}N$-HSQC spectra is typical for membrane helical proteins [62]. Amount of water-exposed amide groups monitored by the CLEANEX spectra suggests that about 20-22 residues of each fragment are located within the micelle. In the bottom of panels **A** and **B**, the CD spectra of the NMR samples are shown in *yellow*. The deconvolution of the CD spectrum of hNEU1/TMS2 revealed the presence of 52.1% of α-helix structure and 5.2%, 19.2% and 23.5% of β-sheet, turn and coil structure, respectively, with NRMSD 0.03%. The deconvolution of the CD spectrum of hNEU1/TMS1 embedded into DPC micelles revealed the presence of 45.6% of α-helix structure and 9.1%, 16.8% and 28.6% of β-sheet, turn and coil structure, respectively, with NRMSD 0.02%. Moderate perturbation of the amide chemical shifts and secondary structure distribution, as compared with DPC micelles, are presumably caused by changes of local environment near the amide groups of the residues and an adaptation of both NEU1/TMS2 and NEU1/TMS1 fragments to increased hydrophobic core and other shape of the Fos-Choline-16 micelles.

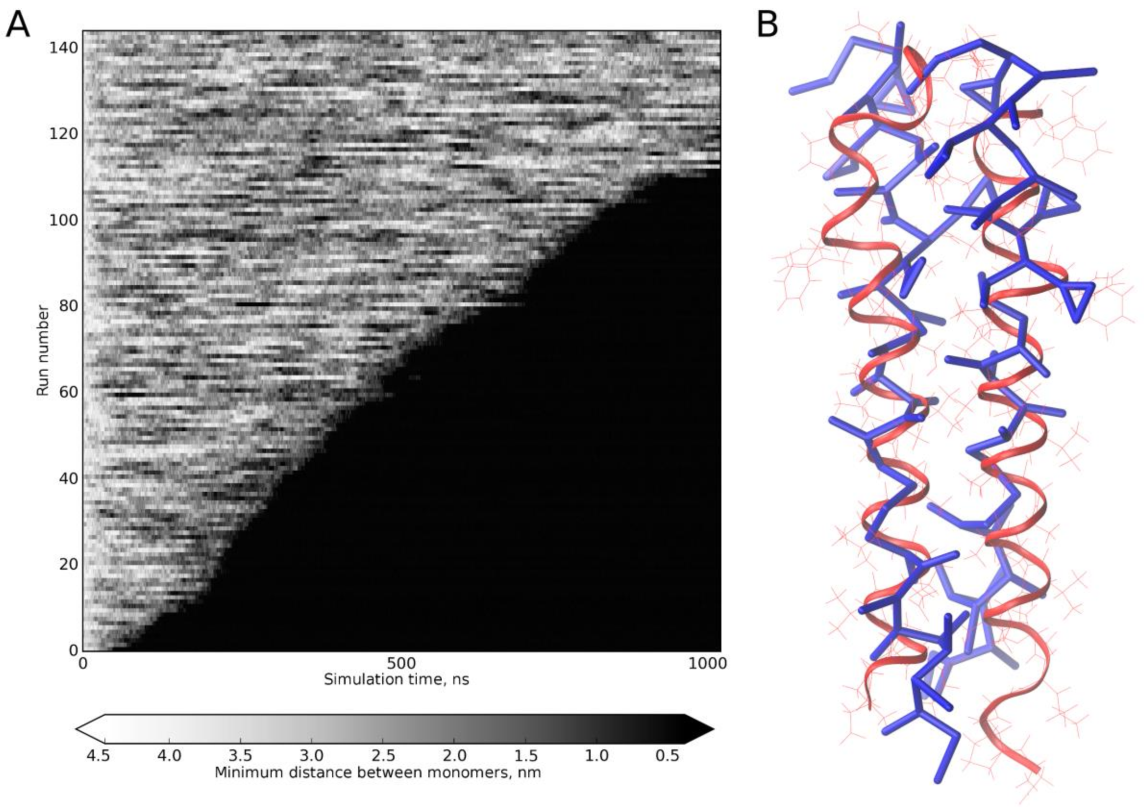

Supplementary Fig. S2 (**A**) Time dependence of spontaneous dimer formation in coarse-grained molecular dynamics (CG MD) simulations of the segment NEU1/TM2 in the POPC bilayer. Minimum distance between the monomers is shown. Darker areas represent smaller distances. 144 MD trajectories are sorted by the dimerization time. (**B**) Ribbon representation of the spatial structures obtained via CG and all-atom simulations. The most populated CG model (blue) is superimposed with the predicted all-atom model 2.

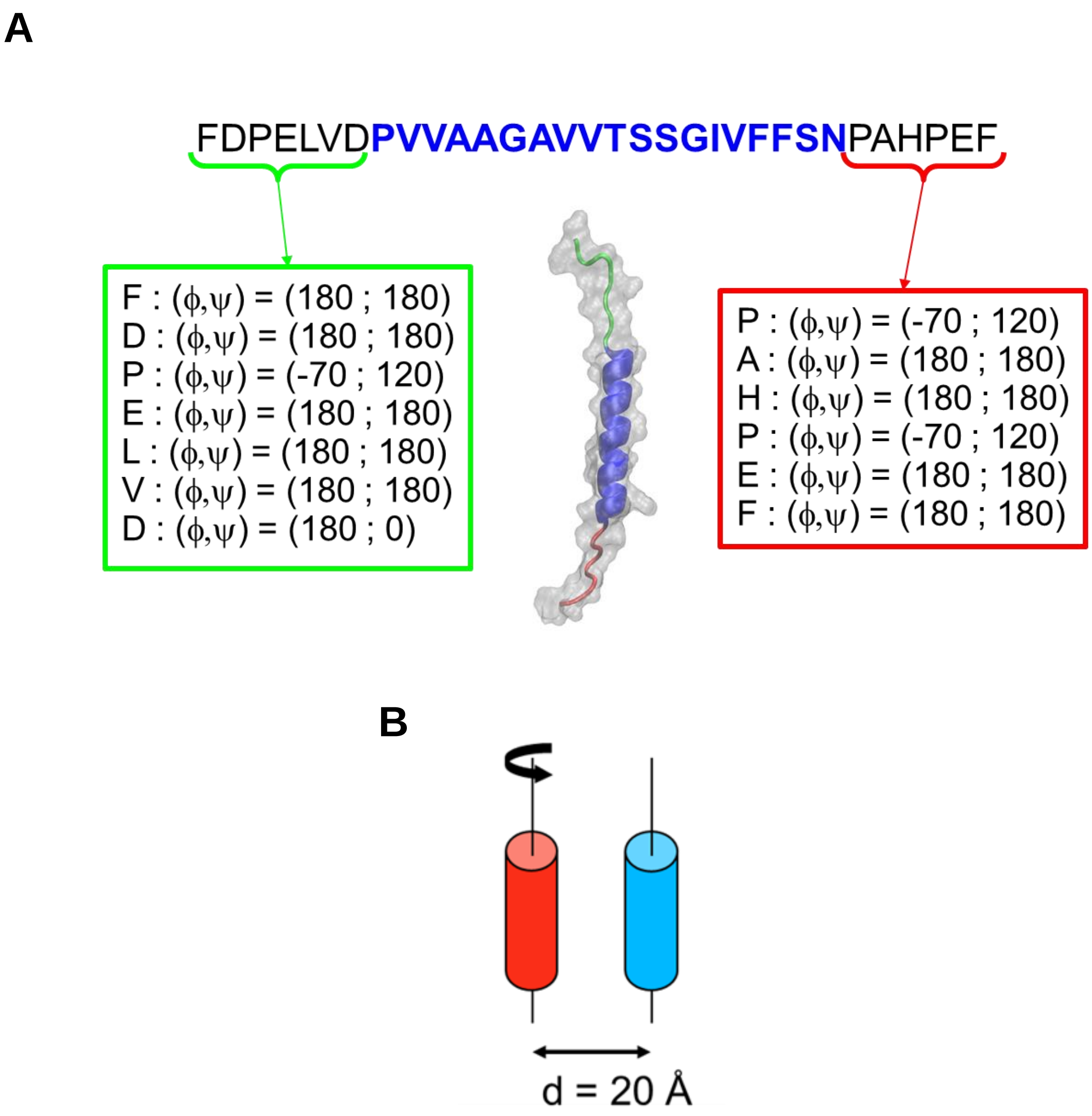

Supplementary Fig. S3 Starting conformations used in MD simulations in DPPC. (**A**) Secondary structure of the modeled NEU1/TM2 peptide. Nineteen residues were placed in an α-helical local conformation (in blue). The conformations of the N- and C-terminal residues (in green and red, respectively) were built in such a way that the termini would point outside the membrane. (**B**) Relative positions and orientations of the two helical peptides. Before being placed in a DPPC membrane, the peptides were positioned in a parallel manner, with a distance of 20 Å between their main axes. The twenty-four different starting conformations were generated using various rotation angles of the red helix around its axis (see the arrow on the figure).

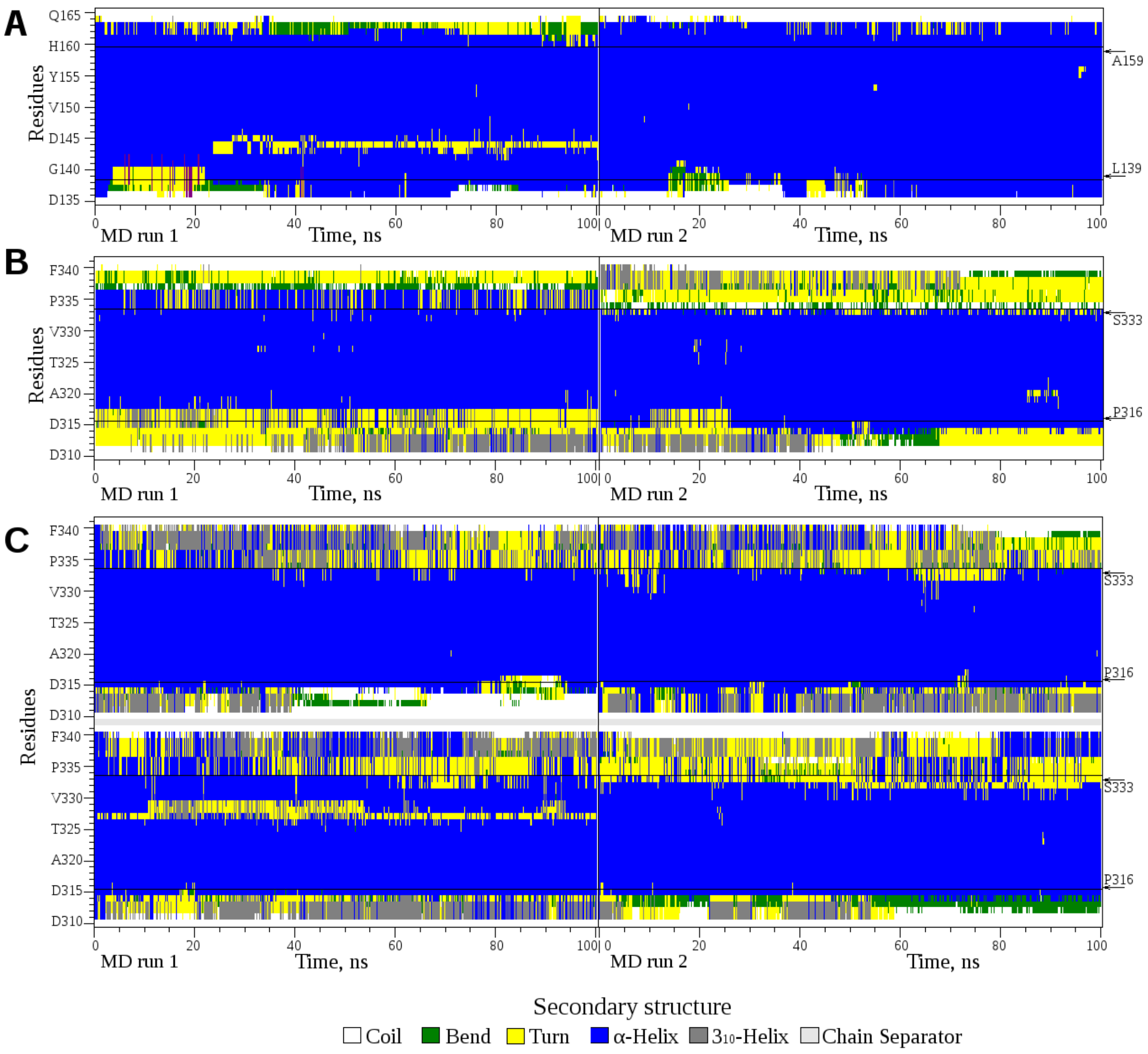

Supplementary Fig. S4 Secondary structure of the peptides during MD simulations in POPC. (**A**) NEU1/TM1 monomers. (**B**) NEU1/TM2 monomers. (**C**) NEU1/TM2 dimers; model 1, left; model 2, right. Colors represent the type of the secondary structure. Amino acid residues are shown on the left. The borders of the TM part are shown with the horizontal black line, with the important residues shown on the right.

Supplementary Table S1 Interfacial residues[$], geometrical parameters, energies and hydrogen bonds for predicted NEU1/TM2 dimer structures.

Model	Inter-helical contacts[$]	Angle deg	D_{HH} (A)	H-bonds[#]	Energy (kJ/mol)
TM2 model 1	DPELVDPVVAA**G**AV**VT**SS**G**IV**F**FSNPAHPEFR	61 ± 3	7.5 ± 0.6	-	-13 ± 5
TM2 model 2	DPELV**D**PVV**A**AGA**V**VT**S**SG**IV**FFS**N**PA**H**PEFR	-3 ± 13	9.6 ± 0.6	S326-S326<br>S333-N334<br>H337-N334	-27 ± 6

[$] In each model, residues on the helix-helix interface are underlined.

[#] Hydrogen bonds between monomers that exist more than 10% of total MD simulation time.
